# Effectiveness of an Energy Management Training Course on Employee Well-Being: A Randomized Controlled Trial

**DOI:** 10.1177/0890117118776875

**Published:** 2018-05-28

**Authors:** Sai Krupa Das, Shawn T. Mason, Taylor A. Vail, Gail V. Rogers, Kara A. Livingston, Jillian G. Whelan, Meghan K. Chin, Caroline M. Blanchard, Jennifer L. Turgiss, Susan B. Roberts

**Affiliations:** 1 Jean Mayer USDA Human Nutrition Research Center on Aging at Tufts University, Boston, MA, USA; 2 Johnson & Johnson, Health and Wellness Solutions Inc, New Brunswick, NJ, USA

**Keywords:** employee wellness program, well-being intervention, behavior change intervention, quality of life, purpose in life

## Abstract

**Purpose:**

Programs focused on employee well-being have gained momentum in recent years, but few have been rigorously evaluated. This study evaluates the effectiveness of an intervention designed to enhance vitality and purpose in life by assessing changes in employee quality of life (QoL) and health-related behaviors.

**Design:**

A worksite-based randomized controlled trial.

**Setting:**

Twelve eligible worksites (8 randomized to the intervention group [IG] and 4 to the wait-listed control group [CG]).

**Participants:**

Employees (n = 240) at the randomized worksites.

**Intervention:**

A 2.5-day group-based behavioral intervention.

**Measures:**

Rand Medical Outcomes Survey (MOS) 36-item Short-Form (SF-36) vitality and QoL measures, Ryff Purpose in Life Scale, Center for Epidemiologic Studies questionnaire for depression, MOS sleep, body weight, physical activity, diet quality, and blood measures for glucose and lipids (which were used to calculate a cardiometabolic risk score) obtained at baseline and 6 months.

**Analysis:**

General linear mixed models were used to compare least squares means or prevalence differences in outcomes between IG and CG participants.

**Results:**

As compared to CG, IG had a significantly higher mean 6-month change on the SF-36 vitality scale (*P* = .003) and scored in the highest categories for 5 of the remaining 7 SF-36 domains: general health (*P* = .014), mental health (*P* = .027), absence of role limitations due to physical problems (*P* = .026), and social functioning (*P* = .007). The IG also had greater improvements in purpose in life (*P* < .001) and sleep quality (index I, *P* = .024; index II, *P* = .021). No statistically significant changes were observed for weight, diet, physical activity, or cardiometabolic risk factors.

**Conclusion:**

An intensive 2.5-day intervention showed improvement in employee QoL and well-being over 6 months.

## Purpose

Over 153 million US civilian adults are employed.^1^ The increasingly poor physical and psychological health of employees is a substantial burden to employers, swelling health-care costs and reducing workforce productivity. Annually, reduced productivity due to depression symptoms alone cost US$44 billion,^2^ while obesity-related absenteeism accounts for another US$10.3 billion.^3^ Nevertheless, adults spend a substantial amount of time at work and employers are stakeholders in employee well-being, which is “a dynamic concept that includes subjective, social, and psychological dimensions as well as health-related behaviors.”^4^ Therefore, employer-based well-being initiatives have unique potential to positively influence physical and psychological health.

Historically, health-related medical expenditures and disability have been the focus of worksite well-being interventions. However, employee retention,^5^ productivity,^6,7^ and engagement^8^ are increasingly recognized as potential programmatic benefits and have resulted in employers embracing interventions to improve psychological health and quality of life (QoL) among employees.^9-12^ Although well-being interventions have been implicated in improving key QoL measures, such as vitality and purpose in life (PiL),^13^ to our knowledge, there has been only 1 randomized controlled trial (RCT) testing the ability of a worksite intervention to positively impact vitality.^14^

The aim of this study was to test whether completers of a 2.5-day intensive intervention—designed to enhance employee health and well-being—would experience improved QoL 6 months later. Our primary objective was to evaluate the intervention’s effects on employee vitality (energy); secondary objectives included effects on other QoL domains, PiL, sleep, mood, and depression, as well as body mass index (BMI) and cardiometabolic risk factors.

## Methods

### Design

This study is an RCT of 12 worksites using a 2:1 allocation in favor of worksites receiving the intervention (n = 8 worksites) versus the wait-listed control condition (n = 4 worksites). Randomization was conducted by a statistician independent from the study, using worksite as the unit of randomization and stratified by employer type (eg, for-profit, nonprofit). The intervention was a 2.5-day employee well-being program developed by the Johnson & Johnson’s Human Performance Institute (J&J-HPI). The study is registered at https://clinicaltrials.gov/ct2/show/NCT02593240 and includes follow-up periods at 6, 12, and 18 months. This report describes the baseline and 6-month follow-up data of the 2.5-day J&J-HPI intervention. All enrollment and study assessments were independently conducted by investigators at the Jean Mayer USDA Human Nutrition Research Center on Aging at Tufts University without involvement of the trial sponsors. The study was approved by the institutional review board of Tufts Health Sciences and written informed consent forms (ICF) were obtained from all participants.

### Sample

A broad range of worksites within the greater Boston area (50-mile radius) were contacted, and using a multistage screening process, the first 12 interested and eligible worksites were enrolled into the study.

#### Recruitment and ICF

Informational sessions detailing the study and randomization were provided at each participating worksite, after which onsite screening and enrollment were conducted. At screening, employees were deemed eligible if they were aged ≥21 years, had a BMI of ≥20 and <50 kg/m^2^, and were willing to sign an ICF, provide their e-mail to receive program materials, complete outcome assessments, and produce a physician release form. Exclusion criteria included remote or contract workers, non-English speakers, pregnancy, mobility limitations, concurrent participation in an intensive lifestyle program, and major diseases, such as active cancer or cardiovascular disease. At each participating worksite, approximately 20 employees were enrolled on a first-come, first-served basis; enrollees at each worksite completed baseline assessments before they were informed about their randomization.

#### Eligibility

To be eligible to participate, worksites had to have been in operation for at least 3 years, have ≥300 employees with a low turnover rate (≤15%), have a postal address, and have contact information for a company representative who was willing to sign a consent form on behalf of their institution, complete a questionnaire for assessment of worksite eligibility, and facilitate employee outreach as well as onsite evaluations conducted by Tufts investigators. Sites were excluded at screening if they had recent, current, or impending onsite, commercially run, well-being programs.

**Figure 1.**
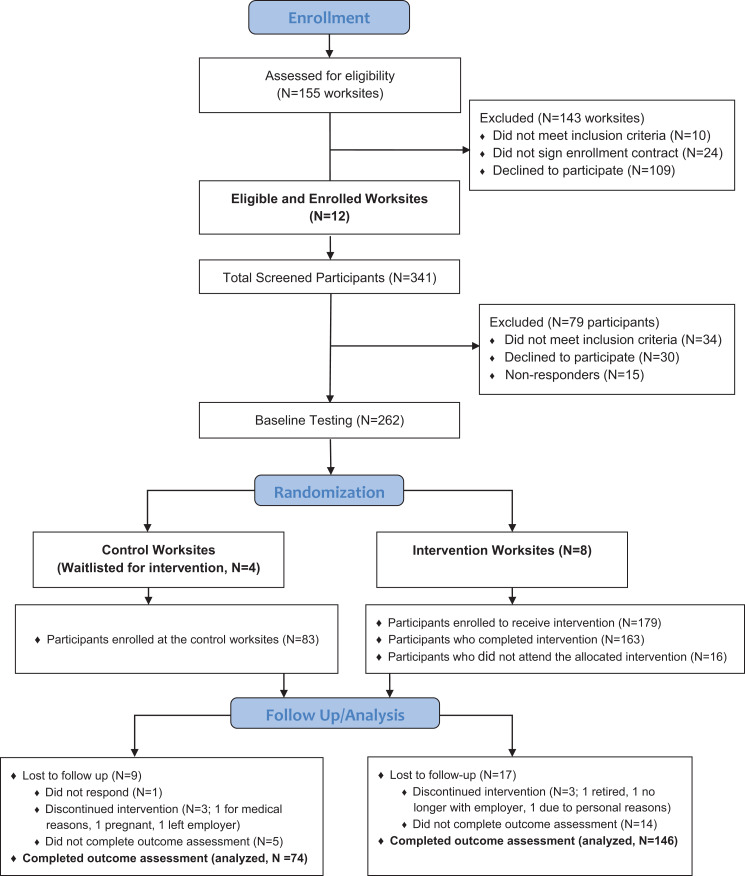
CONSORT chart: Participant enrollment and retention.

As outlined in the Consolidated Standards of Reporting Trials (CONSORT) chart (Figure 1), 155 worksites were recruited between September 2015 and February 2016, 12 of which passed the initial screening questionnaire and were enrolled into the study. Eight worksites (4 universities, 3 for-profit companies, and 1 nonprofit organization) were randomized to the intervention group (IG; 163 participants), while 4 worksites (1 university, 2 for-profit companies, and 1 nonprofit organization) were randomized to the control group (CG; 77 participants). The 2.5-day intervention was provided between February and May 2016, and the 6-month follow-up postintervention was completed between August and December 2016.

### Intervention

The intervention, developed by the J&J-HPI, was delivered by trained coaches as a group-based, in-person employee health and well-being program. The 2.5-day intervention uses a multidisciplinary approach rooted in performance psychology, exercise physiology, and nutrition to help maximize energy and promote lifelong behavior change. To accomplish its aim, the intervention blends cognitive behavioral therapy and acceptance and commitment therapy to directly target the participant’s thoughts, actions, emotional processing, and social interactions.^15-17^ The J&J-HPI team also drew upon clinical experience and the scientific literature at large to develop the intervention’s 2 foundational models: the energy management model and the change process model. According to the energy management model, the program is designed to help employees develop attitudes, knowledge, skills, and behaviors that increase daily energy levels, align with their sense of PiL, and improve their overall functioning in and out of work. Psychologically, the change process model guides participants to establish their own PiL or direction in life, candidly compare their current life with this desired direction, and create an “action plan” for making and sustaining change after program completion.

**Figure 2.**
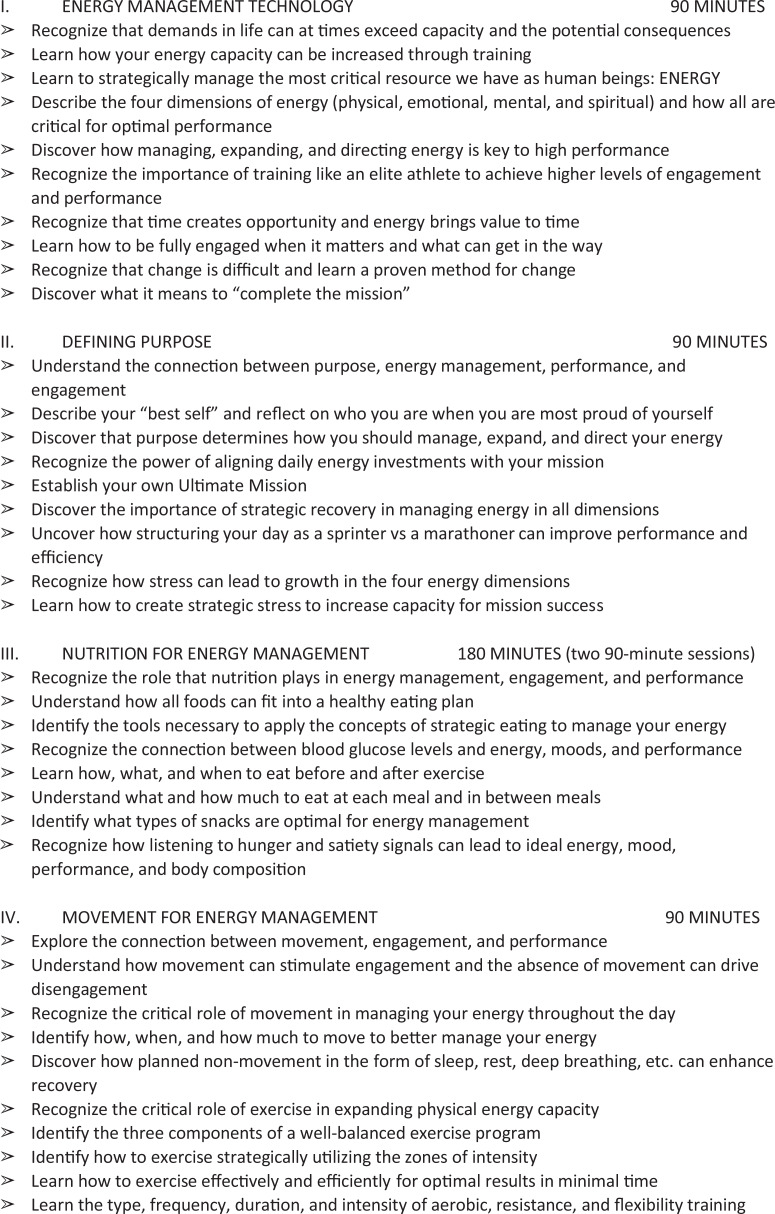
Johnson & Johnson Human Performance Institute 2.5-Day Course Outline.

The immersive intervention was delivered by 3 trained professional coaches over 2.5 days at a venue separate from the employees’ worksite; multiple sessions were offered to accommodate group size and all participants. Participants learned techniques to optimize daily energy levels, create short- and long-term goals, and review feedback from important people in their lives (eg, family and coworkers) through individual reflection, group discussion, didactics, and in vivo exercises (see Figure 2).^18^ Participants who completed the workshop were provided with supplemental educational materials, including the workshop manual, a portable exercise booklet with quick, energizing workouts, and comprehensive online support (e-course) that was made available for the entire follow-up period. These materials encouraged participants to work toward their action plan by adopting behavioral changes aligned with personal goals, such as reducing stress, managing energy, and maximizing purpose.

**Figure:**
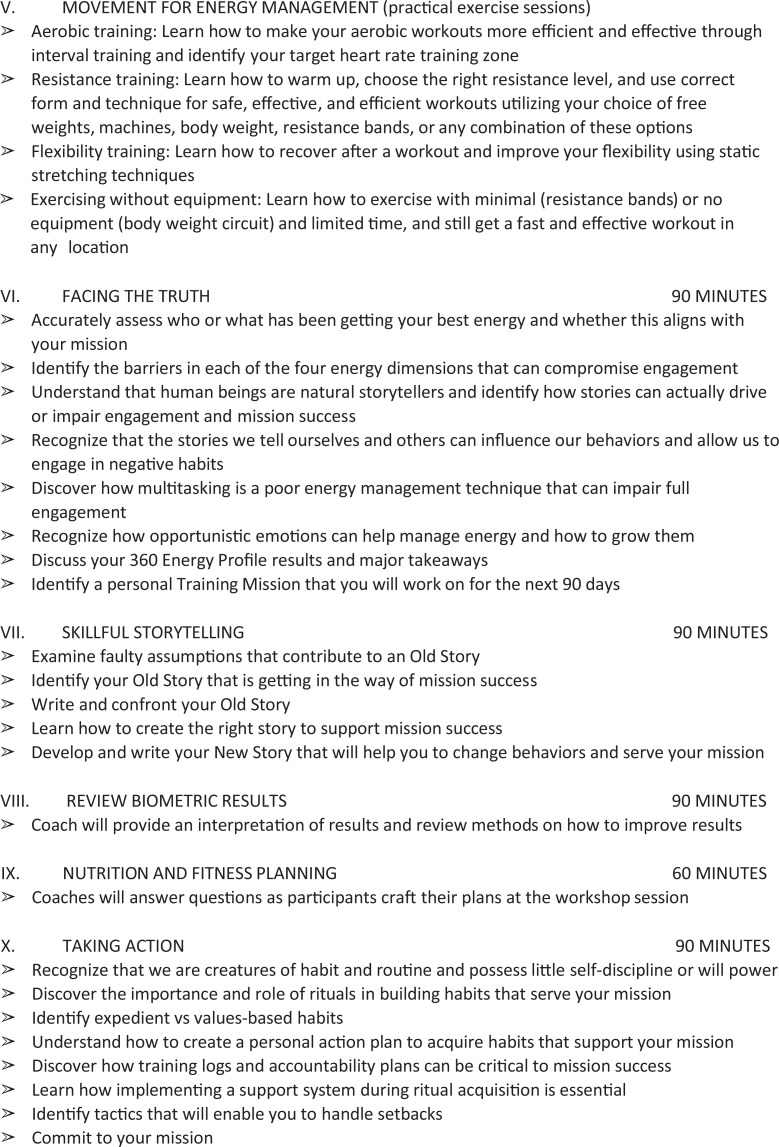


A total of 197 participants from both the IG and CG provided feedback on the 2.5-day workshop. Participants reported high mean ratings for satisfaction (4.7 ±0.7 on a 5-point scale, with 1 being “not satisfied” and 5 being “extremely satisfied”) and likelihood to make significant changes based on the training (4.6 ±0.7 on a 5-point scale, with 1 being “not likely” and 5 being “very likely”).

### Measures

All outcomes were assessed at baseline and 6 months at each of the participating worksites. Self-reported measures were collected by validated questionnaires using an electronic portal (ScienceTrax; Macon, Georgia) with an encrypted identification code unique to the employee. Measures included (a) the Rand Medical Outcome Survey (MOS) 36-item Short-Form (SF-36)^19,20^ consisting of 8 subscales, including vitality (the primary outcome), general health, bodily pain, physical functioning, mental health, role limitations due to physical problems, role limitations due to emotional problems, and social functioning; (b) the 14-item Ryff PiL Scale^21-23^; (c) depression as measured by the Center for Epidemiologic Studies Depression (CESD)^24^; (d) sleep measured using the Rand MOS Sleep Scale; (e) mood using the Profile of Mood States (POMS) questionnaire^25^; and (f) physical activity using the International Physical Activity Questionnaire.^26^

Height was measured only at baseline to ±0.1 cm using a portable stadiometer (seca 213, seca gmbh & co. kg., Hamburg, Germany), and fasting weight (±0.1 kg) and body composition were measured using the Tanita TBF300A (TANITA Corporation, Tokyo, Japan). Waist and hip circumference were measured to ±0.3 cm using seca 201 measuring tape (seca gmbh & co. kg., Hamburg, Germany) and standard procedures. Blood pressure was measured to the nearest 1 mm Hg (3 measurements, 5 minutes apart after 5 minutes of quiet sitting) using the OMRON HEM-705CP digital blood pressure monitor (OMRON Healthcare Co., Ltd., Muko, Japan). Blood samples were collected by a finger stick: Fasting triglycerides, high-density lipoprotein (HDL), low-density lipoprotein (LDL), fasted glucose, and total cholesterol (TC) were measured using the Alere Cholestech LDX system (Alere San Diego, Inc., San Diego, California), and glycated hemoglobin (HbA1c) was measured using the Siemens DCA Vantage (Siemens Healthcare Point of Care Diagnostics, Norwood, Massachusetts).

Sample size was calculated based on the primary outcome (vitality) using an expected 9-point increase^27^ in the IG compared to the CG and a between-worksite standard deviation of 3.4 points. In all, 12 worksites, with a 2:1 allocation in favor of the intervention and 15 participants per worksite, were required to have 80% power to detect a 9-point increase in vitality score.

### Analysis

Data were examined for normality. Baseline characteristics of participants in the IG and CG were described and differences between groups were evaluated using the χ^2^ test for categorical variables and 2-sample *t* tests for continuous variables.

Primary analyses included participants with complete data for the outcome measures. Secondary analyses were performed excluding outliers and utilizing last observation carried forward (LOCF) for missing data. All models were adjusted for the following fixed effects: age (years), sex, ethnicity (white/nonwhite), and baseline value of the outcome of interest. Site nested within intervention status (IG or CG) was classified as a random effect in all models.

For outcomes that were normally distributed, IG and CG were compared by computing least square means and 95% confidence intervals (CI) from general linear mixed models. The main outcomes were the mean change of measures between baseline and month 6 controlling for baseline value. Analyses of cardiometabolic risk factors were additionally adjusted for corresponding medication use and smoking at baseline.

Three change measures for the SF-36 domains were not normally distributed and could not be transformed for analysis. For these measures, cut points were determined for participants who scored in the highest levels of these domains at 6 months; general linear mixed models were used to compare the difference in the prevalence of IG and CG participants in these categories. Least squares means and 95% CIs were calculated for presentation. Significance was determined via a corresponding logistic model to address the binary outcomes. To provide a complete analysis, cut points for all 8 SF-36 domains were created and analyzed in the same manner. The primary outcome, change in vitality, was normally distributed and therefore was analyzed as both continuous and categorical, the latter of which is presented here.

Secondary analysis was performed examining predictors of change in vitality. We computed adjusted least squares means and 95% CIs from a general linear mixed model that included the following measures: intervention status (IG vs CG) and baseline and change values for PiL, sleep problems (indexes I and II), and total physical activity. Models were also adjusted for the covariates previously listed.

Data analyses were performed using SAS version 9.4 (SAS Institute Inc, Cary, North Carolina). All testing was 2-sided, and results with *P* values <.05 were considered statistically significant.

## Results

Table 1 summarizes participant characteristics and baseline values for outcome measures in the IG and CG. Within the enrolled cohort, participants were, on average, 46 years old, female (58.3%), white (77.5%), married or living with a partner (69.6%), and well educated (84.2% reported a college or graduate degree). Also, 65.4% reported annual household incomes ≥US$100,000. Less than 6% self-reported current smoking, high blood pressure, high cholesterol, diabetes, thyroid conditions, or health problems preventing physical activity. Regarding outcome measures, the proportion of employees at risk of clinical depression, defined as a CESD score ≥16, did not significantly differ between IG (22.4%) and CG (27.4%). Significant differences between groups were observed for baseline physical activity level: moderate (*P* = .016), vigorous (*P* = .015), and total physical activity (*P* = .004) were higher in the CG compared to the IG.

**Table 1.** Participant Characteristics at Baseline.

	Control Group (CG), n = 77	Intervention Group (IG), n = 163	*P* Value^a^
Female sex, %	47 (61.0%)	93 (57.0%)	.559
Age, mean (SD), years	45.9 (10.3)	46.7 (11.1)	.564
Hispanic ethnicity, %	7 (9.1)	11 (6.7)	.525^d^
Race, %			
White	62 (80.5)	124 (76.1)	.671
Black/African American	4 (5.2)	8 (4.9)	
Asian	5 (6.5)	19 (11.6)	
Other^b^	6 (7.8)	12 (7.4)	
Marital status, %			
Married or living with partner	53 (68.8)	114 (69.9)	.862
Other^c^	24 (31.2)	49 (30.1)	
Annual household income, %			
US$0-US$59 999	10 (13.0)	11 (6.7)	.144^d^
US$60 000-US$99 999	15 (19.5)	42 (25.8)	
US$100 000+	49 (63.6)	108 (66.3)	
Unknown	3 (3.9)	2 (1.2)	
Highest level of education completed, %			
12th grade/GED, some college/associate’s	10 (13.0)	26 (16.0)	.808^d^
Bachelor’s (includes multiple degrees)	28 (36.4)	63 (38.7)	
Graduate degree (doctoral or nondoctoral)	37 (48.0)	74 (45.4)	
Unknown	2 (2.6)	0 (0.0)	
Current smoker, %^e^	1 (1.3)	8 (4.9)	.280
Ever smoked, %^f^	16 (20.8)	34 (20.9)	.998
Chronic illness, %^g^			
High blood pressure	0 (0.0)	9 (5.5)	.061
High cholesterol	0 (0.0)	2 (1.2)	.999
Diabetes	1 (1.3)	2 (1.2)	.999
Thyroid conditions	0 (0.0)	3 (1.8)	.553
Health problems preventing physical activity, %^g^			
Back problems prevent physical activity	2 (2.6)	4 (2.4)	.999
Foot problems prevent physical activity	2 (2.6)	2 (1.2)	.595
Knee problems prevent physical activity	1 (1.3)	4 (2.4)	.999
Neck problems prevent physical activity	1 (1.3)	1 (0.61)	.540
Asthma prevents physical activity	0 (0.00)	1 (0.61)	.999
Other problems prevent physical activity	2 (2.6)	6 (3.7)	.999
Baseline values for covariates and outcomes measures in this study			
SF-36 health survey measures, mean (SD)^h^			
General health	73.0 (16.0)	68.3 (17.9)	.050
Bodily pain	79.9 (19.0)	80.8 (18.0)	.702
Emotional well-being	73.6 (15.7)	72.5 (15.8)	.603
Physical functioning	92.4 (13.2)	92.9 (11.0)	.739
Role limitations due to emotional problems	75.4 (38.2)	80.4 (33.1)	.309
Role limitations due to physical problems	88.1 (26.6)	89.1 (24.2)	.784
Social functioning	86.7 (19.6)	87.3 (18.2)	.797
Vitality	53.7 (18.7)	53.1 (21.1)	.836
Ryff Purpose in Life Scale, mean (SD)^i^	68.7 (9.2)	65.8 (11.8)	.042
Anthropometric measurements, mean (SD)			
Weight, kg	77.7 (19.3)	78.4 (16.9)	.782
Body mass index	26.9 (5.5)	27.0 (4.9)	.930
Percentage body fat^j^	31.4 (8.2)	31.3 (8.8)	.974
Cardiometabolic risk factors, mean (SD)			
HbA1c, whole blood, %	5.2 (0.4)	5.3 (0.5)	.423
Glucose, mg/dL	95.0 (11.3)	97.1 (14.7)	.243
Total cholesterol, mg/dL	184.7 (36.1)	192.6 (37.5)	.124
Triglycerides, mg/dL	125.4 (100.2)	108.2 (73.0)	.180
HDL, mg/dL^k^	59.0 (20.3)	61.3 (19.8)	.399
LDL, mg/dL^l^	104.2 (32.9)	112.5 (31.9)	.091
Systolic blood pressure, mm Hg	119.3 (15.0)	124.5 (15.0)	.012
Diastolic blood pressure, mm Hg	77.2 (10.5)	79.0 (9.1)	.182
Sleep, mean (SD)			
Sleep problems index I^m^	31.0 (13.3)	29.8 (14.7)	.560
Sleep problems index II^m^	31.9 (13.3)	30.9 (14.9)	.613
Sleep adequacy^m^	46.8 (22.7)	48.1 (24.1)	.703
Sleep disturbance^m^	29.8 (18.4)	27.9 (19.6)	.473
Optimal Sleep scale^n^	0.5 (0.5)	0.5 (0.5)	.735
Sleep quantity^m^	6.6 (0.9)	6.6 (1.0)	.809
Somnolence scale^m^	21.9 (16.0)	23.2 (17.1)	.607
Snoring scale^m^	31.4 (32.0)	30.1 (33.1)	.785
Short of breath scale^m^	6.4 (12.9)	6.7 (14.1)	.887
International Physical Activity Questionnaire (IPAQ), median (IQR)^o^			
IPAQ walking MET, min/wk^p^	693.0 (709.5)	495.0 (726.0)	.220
IPAQ moderate MET, min/wk^q^	360.0 (620.0)	240.0 (480.0)	.016
IPAQ vigorous MET, min/wk^p^	760.0 (1440)	320.0 (1200.0)	.015
IPAQ summary score^q^	2413.5 (1854.0)	1398.7 (1790)	.004
Mood (Profile of Mood States), median (IQR)^r^			
Tension/anxiety^s^	5.0 (5.0)	4.0 (5.0)	.500
Anger/hostility^s^	2.0 (4.0)	2.0 (4.0)	.687
Fatigue^s^	5.0 (7.0)	5.0 (6.0)	.702
Depression/dejection^s^	2.0 (5.0)	1.0 (6.0)	.912
Vigor^t^	10.0 (7.0)	10.0 (5.0)	.550
Confusion/bewilderment^t^	2.5 (4.0)	3.0 (3.0)	.414
Total mood disturbance (summary score)^t^	8.0 (22.0)	7.0 (25.0)	.842
Depression: CESD total score, mean (SD)^u^	10.9 (9.4)	10.2 (8.4)	.565
Percent at risk for depression^v^	20 (27.4)	36 (22.4)	.403

Abbreviations: CESD, Center for Epidemiologic Studies Depression; GED, General Equivalency Diploma; HbA1c, glycated hemoglobin; HDL, high-density lipoprotein; IQR, interquartile range; LDL, low-density lipoprotein; SD, standard deviation.

^a^χ^2^ test for categorical variables and 2 sample *t* test for continuous variables.

^b^Includes American Indian/Alaska Native, multiracial, and unknown/other.

^c^Includes single, widowed, separated, divorced, other/unknown.

^d^Unknowns excluded from *P* value calculation.

^e^CG = 74 and IG = 163; *P* value is for Fisher exact test.

^f^CG = 73 and IG = 155.

^g^*P* value is for Fisher exact test.

^h^CG = 76 and IG = 163.

^i^CG = 76 and IG = 161.

^j^CG = 75 and IG = 162.

^k^CG = 76 and IG = 162.

^l^CG = 65 and IG = 135.

^m^CG = 72 and IG = 162.

^n^CG = 71 and IG = 155.

^o^Significance determined using Wilcoxon 2-sample test (2-sided *P* value). Metabolic equivalent task (MET) expresses the intensity of a physical activity; walking MET = 3.3 × walking minutes × walking days; thus, an individual walking 30 min/d for 7 d/wk would be assigned walking MET = 3.3 × 30 × 7 = 693 MET min/wk. Summary score is sum of MET min/w for walking, moderate, and vigorous activity; IPAQ assigns walking 3.3 METs, moderate activity 4.0 METs, and vigorous activity 8.0 METs.

^p^CG = 72 and IG = 163.

^q^CG = 72 and IG = 162.

^r^*P* value is for Wilcoxon 2 sample test.

^s^CG = 72 and IG = 162.

^t^CG = 72 and IG = 161.

^u^CG = 73 and IG = 161.

^v^Defined as cut point of 16 or greater to identify individuals at risk of clinical depression.

Results from participants completing the intervention are presented here (92.8% of CG and 91.1% of IG enrollees), and analyses with the LOCF were similar and did not alter the statistical significance or direction of the findings (data not shown). There were no statistically significant differences in the baseline characteristics in the dropouts versus completers.

Results for change in outcomes from baseline to 6 months are presented in Table 2, showing changes in the 8 subscales of the SF-36 survey as well as for the PiL measure. At 6 months, IG showed a significantly higher mean change in SF-36 vitality as compared to CG (after multivariate adjustment, 12.65 vs 4.98; *P* = .003). Further, compared to CG, IG showed significantly higher adjusted percentages of participants scoring, on average, in the highest categories for the following SF-36 domains: general health (*P* = .014), mental health (*P* = .027), role limitations due to physical problems (*P* = .026), and social functioning (*P* = .007). Proportions were similar in both groups for physical functioning, and between-group differences were not significant for bodily pain and role limitations due to emotional problems. The adjusted change over time for PiL was significantly higher in the IG than in the CG (*P* < .001), indicating a relative improvement in goals, sense of directedness, feelings of meaning in life, and beliefs that give life purpose.

**Table 2.** Six-Month Change in Perceived Health and Purpose in Life.

	Control Group (CG), n = 74^a^	Intervention Group (IG), n = 146	*P* Value^b^
Adjusted percentages (95% CI) of participants scoring on average in the highest categories at 6 months^c^			
SF-36 health survey measures^d^			
General health	0.5 (0.4-0.6)	0.68 (0.61-0.75)	.014
Bodily pain	0.63 (0.52-0.73)	0.74 (0.66-0.81)	.077
Mental health	0.45 (0.32-0.58)	0.65 (0.56-0.75)	.027
Physical functioning	0.96 (0.91-1.01)	0.95 (0.92-0.99)	.781
Role limitations due to emotional problems	0.81 (0.73-0.89)	0.91 (0.85-0.97)	.106
Role limitations due to physical problems	0.85 (0.78-0.92)	0.95 (0.9-1)	.026
Social functioning	0.81 (0.74-0.88)	0.94 (0.89-0.99)	.007
Adjusted means (95% CI)^c^			
SF-36: vitality	4.98 (1.31-8.66)	12.65 (10.05-15.26)	.003
Adjusted means (95% CI) of 6-month change^c^			
Ryff Purpose in Life Scale^e^	0.27 (−1.49-2.02)	5.22 (3.97-6.48)	<.001

Abbreviations: CI, confidence interval; SF-36, 36-item Short-Form.

^a^74 CG due to 1 control missing SF-36 questionnaire data.

^b^*P* values for categorical analysis computed from logistic regression.

^c^All analyses adjusted for age (years), sex, ethnicity (white/nonwhite), worksite, and baseline value.

^d^Cut points for each measure are as follows: general health ≥75, bodily pain ≥75, mental health ≥80, physical functioning ≥75, role limitations due to emotion ≥66, role limitations due to physical ≥75, social fun ≥75, and vitality ≥80.

^e^74 CG and 143 IG; higher score indicates more goals, sense of directedness, feelings of meaning in life, and beliefs that give life purpose.

Statistically significant reductions in the sleep problems index I (*P* = .024) and index II (*P* = .021) as well as reductions in sleep disturbance (*P* = .013) and higher levels of optimal sleep (*P* = .004) were observed in the IG versus CG (Table 3). No significant differences were observed for other sleep measures, including sleep adequacy, quantity, somnolence, snoring, and shortness of breath. No significant differences were observed for 7 of the 8 POMS domains (anger, confusion, depression, tension, vigor, and summary score); however, the IG reported a significantly greater reduction in fatigue (*P* = .027). The IG also had a larger mean decrease in depressive symptoms (*P* = .042), although at 6 months, there was no significant difference in the percentage of IG and CG participants classified as being at risk of clinical depression (CESD total score ≥16). The change in total activity score from baseline to 6 months did not significantly differ between IG and CG.

**Table 3.** Six-Month Change in Quality of Life Measures.

Quality of Life Measure	Adjusted Means (95% CI)^a^		
	Control Group (CG)	Intervention Group (IG)	*P* Value
Sleep^b^	n = 67	n = 136	
Sleep problems index I	−1.35 (−4.17 to 1.48)	−5.42 (−7.39 to −3.45)	.024
Sleep problems index II	−1.38 (−4.36 to 1.59)	−5.79 (−7.89 to −3.69)	.021
Sleep adequacy	5.08 (−1.11 to 11.28)	7.92 (3.52 to 12.33)	.426
Sleep disturbance	0.02 (−3.43 to 3.47)	−5.63 (−8.04 to −3.21)	.013
Optimal Sleep Scale^c,d^	−0.13 (−0.25 to −0.01)	0.12 (0.03 to 0.2)	.004
Optimal Sleep Scale at month 6^c^	0.35 (0.23 to 0.47)	0.6 (0.51 to 0.68)	.004
Sleep quantity^e^	−0.09 (−0.3 to 0.11)	0.15 (0 to 0.29)	.057
Somnolence Scale	−1.69 (−4.66 to 1.27)	−5.2 (−7.27 to −3.13)	.054
Snoring Scale^f^	−1.77 (−9.29 to 5.74)	−6.73 (−12.14 to −1.32)	.262
Short of Breath Scale	0.89 (−3.32 to 5.11)	−0.62 (−3.6 to 2.35)	.528
Mood (POMS)^g^	n = 65	n = 123	
Summary score	−0.6 (−6.13 to 4.93)	−4.27 (−8.27 to −0.26)	.258
Anger	−0.15 (−1.48 to 1.17)	−0.04 (−1 to 0.92)	.878
Confusion	0.06 (−0.57 to 0.69)	−0.21 (−0.67 to 0.25)	.455
Depression	−0.2 (−1.7 to 1.29)	−0.29 (−1.37 to 0.79)	.920
Fatigue	−0.03 (−1.24 to 1.18)	−1.75 (−2.62 to −0.87)	.027
Tension	0.47 (−0.66 to 1.59)	−0.26 (−1.08 to 0.55)	.265
Vigor	0.86 (−0.24 to 1.96)	1.67 (0.87 to 2.47)	.211
Depression	n = 65	n = 128	
Change in overall CESD score from baseline	−0.14 (−1.82 to 1.54)	−2.28 (−3.5 to −1.07)	.042
Percentage at risk of depression at 6 months^h^	25 (15 to 34)	16 (8 to 23)	.132

Abbreviations: CESD, Center for Epidemiologic Studies Depression Scale; CI, confidence interval; POMS, profile of mood states.

^a^All analyses adjusted for age (years), sex, ethnicity (white/nonwhite), worksite, and baseline value.

^b^Higher sleep quality scores reflect more of the attribute implied by the scale name.

^c^Optimal Sleep Scale response consisted of a yes/no response and, therefore, was not subject to outlying values.

^d^CG = 64, IG = 123.

^e^Sleep quantity had limited values of 4 to 8 hours and, therefore, was not subject to outlier values.

^f^CG = 66, IG = 136.

^g^POMS 65 question version was used; however, the final 11 questions were missing. Domains were calculated excluding missing questions so the ability to compare POMS scores with other populations is limited.

^h^Defined as CESD total score of 16 or higher (less than 16 indicates no risk of clinically significant depression).

Although small decreases in BMI and percentage body fat were observed in the IG, the difference in change over time between the IG and CG was not significant (Table 4). In addition, no significant changes over time were observed between IG and CG for the following cardiometabolic risk factor measurements: HbA1c, triglycerides, LDL, and systolic blood pressure. Fasting glucose and TC increased in both groups; however, the IG showed a much smaller increase over time as compared to the CG (0.03 vs 4.21, *P* = .015 and 0.37 vs 11.06, *P* = .019, respectively). Lower HDL was observed in the IG, while CG showed an increase (−1.99 vs 3.51, *P* = .011). Both groups revealed a reduction in diastolic blood pressure, although the statistical difference was modest (−2.71 vs −0.73 for IG and CG, respectively, *P* = .044).

**Table 4.** Six-Month Change in Anthropometric Measurements and Cardiometabolic Risk Factors.

Anthropometric Measurement/Cardiometabolic Risk Factor	Adjusted Means (95% CI)		
	Control Group (CG), n = 75	Intervention Group (IG), n = 146	*P* Value
Weight, kg^a,b^	−0.03 (−0.73 to 0.67)	−0.43 (−0.92 to 0.07)	.326
BMI^a,b^	0 (−0.25 to 0.24)	−0.16 (−0.33 to 0.02)	.280
Percent body fat^a,c^	0.3 (−0.28 to 0.87)	−0.38 (−0.78 to 0.02)	.058
HBA1C, whole blood, %^d,e^	0.11 (0.04 to 0.18)	0.13 (0.08 to 0.18)	.748
Glucose, mg/dL^d^	4.21 (1.59 to 6.82)	0.03 (−1.8 to 1.85)	.015
Total cholesterol, mg/dL^d^	11.06 (4.05 to 18.08)	0.37 (−4.62 to 5.36)	.019
Triglycerides, mg/dL^d^	10.06 (−5.2 to 25.32)	8.84 (−1.76 to 19.44)	.887
HDL, mg/dL^d,f^	3.51 (0.26 to 6.75)	−1.99 (−4.31 to 0.33)	.011
LDL, mg/dL^d,g^	4.84 (−1.54 to 11.21)	0.75 (−3.78 to 5.27)	.278
Systolic blood pressure, mm Hg^d^	0.85 (−2.42 to 4.11)	−2.39 (−4.73 to −0.06)	.103
Diastolic blood pressure, mm Hg^d^	−0.73 (−2.31 to 0.84)	−2.71 (−3.81 to −1.61)	.044
Metabolic syndrome at month 6, %^d,h^	30.4 (22.9 to 38.0)	26.9 (21.6 to 32.2)	.416

Abbreviations: BMI, body mass index; CI, confidence interval; HbA1c, glycated hemoglobin; HDL, high-density lipoprotein; LDL, low-density lipoprotein.

^a^Adjusted for age (years), sex, ethnicity, worksite, and baseline value.

^b^CG = 73 and IG = 146.

^c^CG = 69 and IG = 141.

^d^Adjusted for age (years), sex, ethnicity (white/nonwhite), smoking at baseline (yes/no), medication use, worksite, and baseline value. Medication use was defined as glucose-lowering medication for HBA1C and glucose models, cholesterol-lowering medication for total cholesterol, triglycerides, HDL, and LDL models, and blood pressure-lowering medication for systolic and diastolic models. Positively skewed variables were examined on both original and logged scales with similar results. Original data are presented.

^e^CG = 75 and IG = 145.

^f^CG = 74 and IG = 145.

^g^CG = 58 and IG = 110.

^h^Based on the ATP 3 guidelines of having 3 or more of the following: waist circumference of >102 cm for men and >88 cm for women, fasting plasma triglycerides ≥150mg/dL or taking cholesterol-lowering medication, fasting HDL cholesterol <40 mg/dL for men or <50 mg/dL for women, or taking cholesterol-lowering medication, systolic blood pressure ≥130 mm Hg and/or diastolic blood pressure ≥85 mm Hg, or taking hypertension medication, fasting plasma glucose ≥100 mg/dL, or taking diabetes medication.

Predictors of change in vitality showed that the intervention remained a significant predictor of positive change in vitality (IG = 11.67 vs CG = 7.1, *P* = .038). Baseline vitality and sleep problems were inversely associated with vitality change (*P* < .0001 and *P* = .004, respectively), while improvements in sleep (*P* = .0009) as well as baseline and enhanced PiL (*P* = .005 and *P* < .001, respectively) were all positive predictors of change in vitality. No other measures were statistically significant.

## Discussion

Employee health and well-being are important determinants of workforce productivity and engagement^28,29^ and substantially impact health-care costs.^2,3^ The findings from this RCT of a 2.5-day immersive well-being intervention across 12 diverse worksites demonstrated significant improvements in employee vitality (energy) and PiL, as well as self-reported general health, mental health, social functioning, and emotional and physical role limitations. There were also significant improvements in sleep, fatigue, and depression symptoms. To our knowledge, this is the first study to demonstrate significant improvements in multiple QoL metrics with a worksite-based intervention in employees.

Within the broad categories of QoL and well-being, vitality and PiL were defined as primary variables because they reflect fundamental aspects of existence and enhancement of life with purpose, which provide direction and the energy to support QoL.^18^ The importance of these measures as the key factors of QoL, health, and well-being has only recently received attention in the context of worksite well-being programs. For example, van Steenbergen et al^30^ showed that vitality was significantly associated with motivation, absenteeism, presenteeism, health care, and work performance. A growing body of evidence also demonstrates that PiL is tied to psychological health,^31^ biological health indicators,^32^, longevity,^33^ preventative self-care,^34^ and health-care utilization metrics, such as length of hospital stays.^14,34^ Furthermore, higher PiL is associated with lower risk of Alzheimer disease and mild cognitive impairment as well as risk of most noncommunicable diseases^35-38^ and premature death.^39^ With a rapidly aging workforce and concomitant increases in health-care costs, interventions focusing on vitality and PiL may be particularly beneficial for maintaining and optimizing employee well-being. It is also noteworthy that the reported improvements in sleep and general health with the intervention occurred in the absence of marked changes in measured cardiometabolic risk factors, implying that mental well-being can be improved without changes in physical health. However, as physical health has independent effects on health-care costs, the type of intervention tested herein ideally would be combined with interventions aiming to improve physical health.

A key strength of this study is the methodological rigor used to address criticisms that are common in most worksite interventions and that often influence biases and study conclusions, particularly in studies of psychological health and well-being.^9,29,40,41^ These include lack of randomization and failure to include a CG or follow-up period.^40^ Furthermore, worksite wellness RCTs that previously attempted to address these limitations were unable to clearly demonstrate a positive effect, often due to high attrition.^41-43^ In a recent systematic review of mental health and wellness interventions conducted in organizational settings, methodological quality was evaluated using the National Institute for Health and Care Excellence (NICE) guidelines, and 10 of the 11 studies were identified as having high risk of bias, particularly with regard to selection, performance, attrition, and detection biases.^40^ Our study adhered to the NICE guidelines, with no attrition in the worksites randomized to the IG or CG, suggesting a very low risk of bias. The CG participants, possibly due to the anticipation of receiving the intervention at the end of the 6-month period, had a slightly lower attrition rate than IG participants.

### Limitations

The self-selected worksites and use of self-reported measures are possible limitations in this study. However, the inclusion of a CG may mitigate potential biases.

**So What?**

**What Is Already Known on This Topic?**

Poor physical and psychological status of employees negatively impacts employer health care and productivity. Adults spend a substantial amount of time at work and employers are stakeholders in the well-being of their employees; therefore, employer-based initiatives have unique potential to improve overall well-being in the workplace.

**What Does This Article Add?**

Although programs focused on employee well-being have gained momentum in recent years, few have been rigorously evaluated for broad implementation in diverse workplaces. Using an RCT design for testing program efficacy, our study found that, 6 months after completing an intensive 2.5-day intervention, employees from diverse workplaces experienced improved vitality (energy), QoL, PiL, and sleep.

**What Are the Implications for Health Promotion Practice or Research?**

Studies in the workplace are critical for examining the true effect and potential value of workplace interventions. However, the implementation and testing of workplace interventions present serious logistical and methodological obstacles, including organizational structure, business objectives, and demands on resources. Although there is a continued focus on employee health and well-being, high-quality studies that rigorously examine the specifics of psychological interventions (eg, QoL measures and overall effectiveness) are somewhat limited.^40^ Our findings suggest that well-being programs, such as the one examined here, may be used not only to enhance employee psychological well-being but also to supplement other health-related interventions. Additionally, these studies could determine whether the psychological improvements observed 6 months after the intensive well-being workshop could be sustained further and possibly extend to physical health. Our findings also support future studies of varied duration on this and similar employer-based well-being initiatives to measure intensity, sustainability, and frequency of delivery and touchpoints, all of which could help us better understand how to maximize participation, cost-effectiveness, and benefits of the program.

## Supplemental Material

Supplemental Material, Das_Protocol - Effectiveness of an Energy Management Training Course on Employee Well-Being: A Randomized Controlled Trial Click here for additional data file. Supplemental Material, Das_Protocol for Effectiveness of an Energy Management Training Course on Employee Well-Being: A Randomized Controlled Trial by Sai Krupa Das, Shawn T. Mason, Taylor A. Vail, Gail V. Rogers, Kara A. Livingston, Jillian G. Whelan, Meghan K. Chin, Caroline M. Blanchard, Jennifer L. Turgiss, and Susan B. Roberts in American Journal of Health Promotion

## Supplemental Material

Supplemental Material, Permission_Request-Corporate_Athlete_Outline1_(1) - Effectiveness of an Energy Management Training Course on Employee Well-Being: A Randomized Controlled Trial Click here for additional data file. Supplemental Material, Permission_Request-Corporate_Athlete_Outline1_(1) for Effectiveness of an Energy Management Training Course on Employee Well-Being: A Randomized Controlled Trial by Sai Krupa Das, Shawn T. Mason, Taylor A. Vail, Gail V. Rogers, Kara A. Livingston, Jillian G. Whelan, Meghan K. Chin, Caroline M. Blanchard, Jennifer L. Turgiss, and Susan B. Roberts in American Journal of Health Promotion
