# Supplementary material for: Effectiveness of an Energy Management Training Course on Employee Well-Being: A Randomized Controlled Trial
Source: Am J Health Promot. 2018 May 28;33(1):118–30. doi: 10.1177/0890117118776875 (PMC7323760; doi:10.1177/0890117118776875)
Supplement: Supplemental Material, Das_Protocol - Effectiveness of an Energy Management Training Course on Employee Well-Being: A Randomized Controlled Trial [file Das_Protocol.docx]

**TUFTS MEDICAL CENTER/TUFTS UNIVERSITY RESEARCH PROTOCOL**

**Version date: 7/21/2017**

**Principal Investigators:** Sai Krupa Das, PhD (IRB-PI) and Susan B Roberts, PhD

**Co-Investigator:** Cheryl Gilhooly, PhD

**Study Physician:** Edward Saltzman, MD

**Study Title:** Worksite Wellness Study

1. **Aim and Hypotheses**

Worksites offer attractive locations for reducing the national prevalence of overweight and obesity. Interventions that are both effective and sustainable in producing long-term changes in health and employee wellness are urgently needed. In an 18-month worksite randomized controlled trial in 12 worksites (defined as whole, moderate sized, worksites or subunits of larger worksites) we propose the testing of two lifestyle approaches that are aimed at behavior modification for achieving long-term improvements in health and quality of life. Worksites randomized to the immediate intervention arm will receive the two wellness programs, and the randomized control sites will participate in outcomes assessments only for the initial 6 month period after which participants will be able to participate in the program of their choice. The multiple-program intervention will be tested against the control in a variety of employment types to determine effectiveness for quality of life, health and weight-related wellness across a broad range of demographics. The study will be officially registered as a clinical trial with the intention of submission to a top medical journal, and will be referred to as the Worksite Wellness Study.

The primary focus of the Worksite Wellness Study is to improve health related quality of life, with a particular focus on a decrease in cardiometabolic risk factors, improved weight-related wellness, and improved energy in work and life.

We plan to test our central hypothesis and accomplish the study objective with the following technical objectives:

1. **Conduct a randomized controlled trial assigning worksites in a 2:1 ratio to a multiple-program health intervention or a wait-listed control.** The multiple-program intervention will be a combination of the Human Performance Institute (HPI) intervention and iDiet, a behavioral weight loss program, with randomization to either partial provided food or a gift card for self-selection of food. Within worksites, employees will be eligible to select either the HPI intervention or the behavioral intervention. **Primary outcomes will be quality of life and energy levels (HPI participants and controls) and weight (weight loss intervention participants) at 6-18 months.** Secondary outcomes include cardiometabolic risk factors.
2. *The HPI working hypothesis* is that enrollment in the HPI intervention will result in significant improvements in quality of life (primary outcome) and energy levels compared to controls over 6 months and no significant decrease in quality of life during the subsequent 12-month period in the intervention participants. Exploratory outcomes also tested will include body weight and fat, fasting blood glucose, a lipid panel and systolic and diastolic blood pressure.
3. *The behavioral weight loss program working hypothesis is that* iDiet participants receiving gift card will lose weight over the first 6 months compared to weight listed controls and will lose weight compared to baseline over 18 months. In addition, iDiet participants receiving the ready-prepared foods will lose more weight over 18 months compared to participants receiving gift cards.
4. An exploratory hypothesis is that greater weight loss will be associated with reduced levels of hunger and food cravings and increased satiety.
5. **Background and Rationale**
   1. Background

Currently, one hundred and forty million people aged 20 and older are employed in the United States (1). A variety of health risks ranging from stress and lack of emotional fulfillment to obesity and physical inactivity have been correlated with a reduction in work productivity (presenteeism) (1-3). Not only do these health risks increase presenteeism in the workplace, they also increase health care costs, absenteeism, workplace injury and worker’s compensation claims (1, 3-5). Furthermore, of those currently employed in the United States, 29% of them are obese (1). Stress at work, job insecurity, lack of social support and social eating have been cited as significant contributors to this obesogenic work environment, which is on track to increase to 50% by 2030 (6-10). In an analysis of the National Health and Nutrition Surveys and the National Health Interview Survey, obese workers had the highest prevalence of weight- related diseases compared to normal weight workers, and the rate of metabolic syndrome among obese workers was 53.6% compared to 5.7% among those with normal weight. The metabolic disorders precipitated by excess body fatness are also widespread (11) and, in particular, obesity significantly increases the risk of developing cardiovascular disease, type 2 diabetes mellitus, some cancers and various skeletal disorders such as osteoarthritis (1,11,12).

As expected, there are substantial health care and other costs associated with reduced employee wellness (13). Thompson et al. (14) reported that increased expenditure of worksites for excess medical payments associated with obesity in the U.S. are $7.7 billion, costs for additional sick leave are $2.4 billion, and extra life insurance payments and disability payments are $2.6 billion. Tucker et al. (15) estimated that current health care costs for working obese individuals are over $1,000/year, which is greater than for non-obese working individuals, even ignoring the greater potential for future co- morbidities. Treatment of obesity and ongoing prevention of weight regain in worksites, if effective, holds the potential of being cost-effective when reductions in productivity and increased health care expenses for an overweight population are taken into account.

Since the worksite is an environment where people spend considerable amounts of time on a regular basis, they have been identified as an ideal forum to facilitate healthier lifestyle behaviors (16-25). Employees working together may have a positive influence on each other’s behaviors (18, 26), and from an ecological perspective, behaviors adopted by individual employees may also impact their families and communities (27). Some examples of previous worksite interventions that were successful either in making environmental changes or improving health behaviors include the Seattle 5 a Day program (28) to increase fruit and vegetable consumption; Take Heart II (29-30) to reduce smoking, decrease fat and increase fiber intake, the Next Step Trial (31) to decrease dietary fat and improve fiber consumption among auto workers through nutrition classes; the Working Well Trial (32) that improved the nutrition environments; the Working Healthy Project (33) that improved diet and physical activity behaviors and the Health and Promotion Program (23) to decrease health risks and rates of absenteeism. Additionally, research has shown that the provided foods may modify the environment presumed to influence eating behaviors, and therefore may be a useful strategy for promoting and enhancing weight-loss (34, 35).

The proposed research will implement a randomized controlled trial of two very different worksite wellness programs aimed at promoting wellness and health related quality of life, one with a particular focus on improvements in reported energy levels and the other focused on weight control. The project brings together our expertise in worksite nutrition studies, community weight loss programs and high quality health outcomes. The programs being tested differ in emphasis, approach and delivery, of the health-related wellness they recommend, and by combining them worksites potentially can reach a broader audience than if only one program was offered. The HPI program focuses on energy management and uses the sciences of performance psychology, exercise physiology and nutrition, while the iDiet weight management program emphasizes specific dietary composition goals based on recent promising weight control research by us and others (36-40) and will include an arm with provided foods. Additionally, the study will also examine whether there is a “ripple effect” of the benefits of the interventions from the worksite participants on their co-workers. This latter novel component of our study is based on research by us and others (18, 26, 27, 36, 38) suggesting that successful weight control interventions benefit both the direct participants and the household members who live with them and/or their work colleagues. For example, in a recent study by our team, weight changes were -1.3 ± 0.7kg, respectively (p=0.03) for individuals associated with individuals participating in an intervention versus the individuals associated with wait-listed controls (36). The likely explanation for this effect is that shared meals and exercise habits lead to favorable changes in energy balance in the non-participating adults, an effect that is also anticipated in worksites and, if proven here, could provide a new approach to helping resolve the obesity epidemic in worksite personnel themselves.

The interventions that are to be used in this study are all long-term group behavioral wellness programs, and implementing them simultaneously allows for a combined evaluation of these programs for concurrent use in the workplace. The combination of the 2 programs is anticipated to be highly acceptable to worksites because they are commercially available for large scale dissemination and delivery in a group format. It is anticipated that both of these programs may be beneficial in affecting wellness..

- 1. Rationale

The rationale for these technical objectives is that there is an urgent need for sustainable worksite wellness interventions generally, and two successful programs designed to achieve worksite wellness and improvements in employee health are being tested for sustainability in this long term study. Preliminary data suggests that HPI may be particularly effective in improving overall energy, as well as general and mental health, while our prototype iDiet intervention may be particularly effective and sustainable for long-term improvements in weight. Furthermore, the provision of foods for a subset of participants in the iDiet arm may facilitate adherence and overall weight control, suggesting that provided foods offer an important adjunct to enhance effectiveness of worksite weight loss programs. By conducting a comparison of the more specific dietary composition profiles in the iDiet to the energy maintenance focus of the HPI intervention we will be evaluating current best practices for an extended period of time (18 months) at worksites. The intention is to help all employees and worksites participating in this research program and to identify the most optimal long term approach to wellness, involving dietary and exercise recommendations as well as particular food provisions necessary for sustainable health. Once the results of this study are available, the most effective long-term approach to health and wellness/weight control can then be used for scaling practical, effective programs for worksite wellness.

1. **Research Plan**
2. **Experimental design:** 18-month worksite randomized controlled trial, in which worksites are randomized to receive the multiple intervention wellness programs or the control program. Control participants will participate in outcomes assessments only for the first 6 months, after which they will enroll in the intervention of their choosing. Both arms (HPI and the iDiet) will receive direct training from the coaches who deliver these commercial programs at their respective worksite and/or via a concurrent online session, or at an alternative commutable location that is offsite in the case of the HPI program. A total of 400 participants in the worksites allocated to the intervention, with approximately 20 participants in the HPI arm and 30 in the iDiet arm per worksite is planned to ensure even balance in each of the study arms. If more than 20 individuals are interested in and eligible for the HPI arm or more than 30 are interested in and eligible for the diet arm, participants will be enrolled into the study on a first-come, first-serve basis, including a small upward or downward adjustment in the total number of employees enrolled per site to ensure that the overall targeted number of participants is achieved. All participating individuals, from both the intervention wellness and control programs, can opt-in to either the HPI or the iDiet weight loss program. However, if they choose to participate in the weight loss intervention they will then be randomized into one of two groups: iDiet with food provided or iDiet with a gift card,. The study flow and primary intervention groups are described below:

**
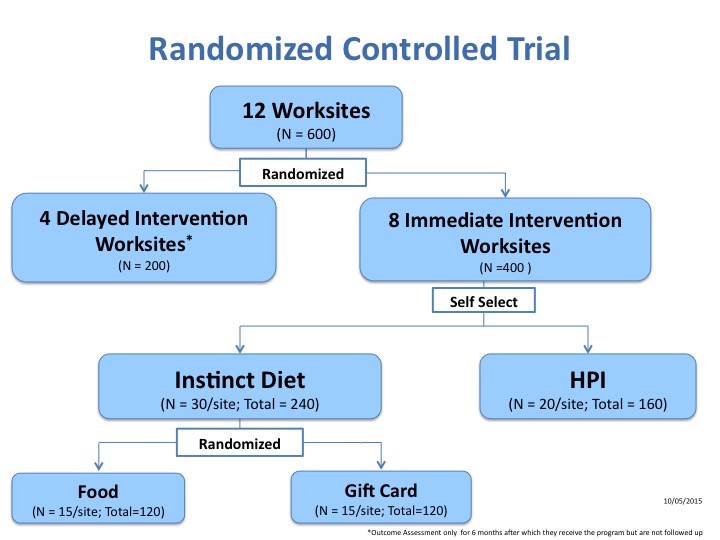
**

*^1^The number of controls reflects study design in which sign up for the intervention and distribution of the intervention sites happens before baseline to ensure equivocal disposition*.

- Group 1: Human Performance Institute (HPI) training workshop: Is comprised of a comprehensive individual development training program that focuses on the concept of energy management. The training includes sections on a conscious evaluation of one’s goals in life, tips to manage and improve personal energy to achieve goals, as well as sessions on nutrition and exercise. The primary aim of this program is to help individuals develop attitudes, habits, and behaviors that increase their levels of daily energy, life satisfaction, and overall functioning. The course is delivered over 2.5 days in a group format with 25 – 32 participants per group by 3 coaches, each of whom specializes in one of the areas described above. Online support modules and supporting educational materials will be available to participants for the remaining 12 month period.
- Group 2: Intensive iDiet group behavioral weight loss program delivered in-person at the worksite or by videoconference and with support of the program’s web platform; This intervention will be delivered by trained nutritionists and will consist of 1-hour support and education meetings of approximately 15 participants/group with weekly messages via the iDiet message board for individual support. The 1-hour meetings will be weekly for 24 weeks (the “active weight loss” period) and then reduced to approximately once a month for the remainder of the 18-month trial. Additionally, following the weekly classes online support will also be provided for the remainder of the study (“structured maintenance” period) with additional booster classes provided on an as-needed basis. The intervention involves a diet behavioral program with particular emphasis on hunger reduction and craving control for both weight loss and prevention of weight regain.
- **Yevo Foods:** Participants will be given commercial dried food products from Yevo Intl., which are designed to help with weight control and health. Of the participants selecting the iDiet, half the group will be randomized to receive Yevo foods. Participants will be supplied with these foods monthly at the worksite for the first six months (equivalent to 2 meals/day during the first 12 weeks, and 1 meal/day for the following 12 weeks). No meals will be provided after this period, however weight maintenance in participants randomized to receive the meal will be examined for over the 18 month study duration. Participants will be asked to communicate directly with research staff for questions about Yevo foods and food vouchers, not with iDiet staff.
- **Gift Cards:** The remaining half of the iDiet participants will be randomized to receive gift cards monthly for the first 6 months, for a total of $378 and will be encouraged to purchase foods at their local grocery store.

1. **Sample size and statistical analyses**

Primary randomization is at the level of the worksites using a 2 to 1 allocation in favor of intervention; 8 worksites will receive the intervention package consisting of the 3 wellness programs and 4 worksites will be randomized as wait-listed controls.

We will enroll up to 700 individuals among 12 worksites with the goal of having 600 complete baseline assessments and get randomized to their intervention. 400 will receive one of the two intervention arms at the intervention sites, while 200 participants will be in the control sites and will participate in outcome assessments only for a six month period.

Primary outcomes measured in this study will be the change in (energy levels) vitality score as measured by the SF-36 questionnaire and change in weight from baseline to 6, 12 and 18 months. Vitality will be the primary outcome in participants in the HPI arm of the study, while weight will be the primary outcome in participants in the behavioral weight loss arm of the study.

The increase in vitality score in the intervention worksites compared to controls is expected to be 9 points at both time points compared to the control. Since randomization is at the worksite level, the total variance includes both the within worksite and between worksite variance. Based on a within worksite standard deviation of 15 and 20 people per worksite and a between worksite standard deviation of at most 3.4, 12 worksites with a 2:1 worksite allocation in favor of treatment are needed to have 80% power to detect an expected increase in vitality score of 9 points.

For the participants electing to participate in the behavioral weight loss program, randomization to either with provided Yevo foods or a gift card such that one half of the participants are in one group and the other half will be assigned to the remaining arm.. Assuming worksite is a fixed variable, 8 worksites with 30 subjects each (240 total subjects) is at least 80% powered to detect a 2.4% difference in weight loss with provided food compared to gift cards assuming no diet by food interaction. If there is a significant food by diet interaction, the study will have an 80% power to detect an interaction that is a 4.5% difference in weight change with provided food for iDiet and 3% improvement in diet.

Data analyses will be performed using the latest version of SAS for Windows, currently 9.4 (Cary, NC: SAS Institute Inc.). All testing will be two-sided and results will be judged statistically significant if the observed significance level (P value) is <0.05. Data will be examined graphically and numerically for outliers and other aberrations that might lead to misleading results. Data may be transformed prior to formal analysis if necessary to fit the assumptions for the statistical model to be employed. Both studies (HPI intervention and the iDiet intervention) will be analyzed according to Intention-To-Treat principles to best reflect what happens when individuals start one of the interventions. If necessary, missing data will be estimated using multiple imputations using the MI Procedure of SAS for Windows.

For the HPI intervention in whom those randomized to intervention will be compared primarily with subjects at control sites who elected the HPI intervention prior to workplace randomization, a mixed linear model with worksite nested within the randomization to HPI intervention or control as a random factor will be use to assess the effect of the intervention on change in vitality scale score. Baseline vitality score, weight, ethnicity and gender will be included as covariates.

The diet intervention will compare those receiving the iDiet with a primary outcome of percent change in weight at 6 and 18 months. To assess the effect of diet and food delivery method, a multi-factor model with diet, food and worksite as independent variables will be used, controlling for baseline weight, gender and ethnicity. Interactions between diet and worksite, food and worksite and food and diet will be assessed and included in the final model if significant. Change in weight for the HPI intervention andchange in vitality score for those in the diet intervention will be included as secondary outcomes.

Secondary analyses will include a comparison of the 3 intervention arms (with and without provided food for the iDiet) as well as the combined effect of intervention on change in anthropometric measures including body fat, and also lipids, blood glucose levels and blood pressure using the same models described above adjusting for the appropriate baseline values. Additionally, regression models will be used to assess the effect of different adherence metrics, activity levels, appetite regulation (hunger and food cravings) and measures of wellbeing on outcome measurements both within and between intervention groups for each arm of the study. In addition, predictors of changes in cardiometabolic parameters will be examined. Prior to enrollment of subjects, a formal data analysis plan will be prepared and submitted as part of clinical trial registration.

1. **Worksite Characteristics**

A convenient sample of worksites in the Greater Boston area, consisting of worksites which have previously expressed interest, and respond to the recruitment letters will be invited to participate in the Worksite Wellness Study. The first twelve eligible worksites with a higher employee interest in worksite wellness will then be invited to enroll in the study.

**Summary of eligibility requirements for worksites to participate**

1. 300 or more employees from a for-profit or non-profit company and/or government institution (including schools, government agencies).
2. Available address and/or email contact for HR/company representative.
3. After understanding the study purpose, HR/company representative and administration’s willingness to be randomized to control or intervention group, and to host onsite and/or online iDiet weight loss groups and facilitate the implementation of the HPI workshop including allowing the interested employees to participate on the assigned dates.
4. Does not have an active weight loss/wellness intervention program onsite currently or within past 6 months or a pending contract for implementation in the immediate future (defined as weekly behavioral groups run by an outside commercial company for ≥14 weeks per year).
5. Low employee turnover rate (approximately 15%)
6. Company has been in operation for at least 3 years
7. HR/company representative can provide the Tufts team with aggregate demographic information (age range of employees, number of males and females etc.).
8. HR/company representative is willing to sign a consent form agreeing to company participation; also, HR/company representative is willing to complete questionnaire to allow preliminary assessment of eligibility, and then send around information and request employees to complete screening questionnaire (or provide emails to Tufts team to do this).
9. HR/company representative is supportive of the study, anticipating that employee enrollment in the program arms will not be problematic (approximately 50 total employees), and are able post informational flyers and study information for employees around the worksite and send occasional reminder emails to all employees prior to the two health surveys (or provide emails to Tufts team so that they can make these contacts).
10. There is a room available for the Tufts team to lead seminars and groups at times that are convenient to the worksite. Also, a bulletin board available for study flyers, and central location for distribution of study handouts and setting up periodic booth for information.
11. **Subject Characteristics**
    - 1. Inclusion criteria:
         1. Willingness to sign an informed consent form
         2. ≥ 21 years of age
         3. Willingness to provide release from his/her physician before starting the intervention program-a requirement for each of the 3 wellness programs which can be faxed to 617 556 3133, or emailed to [worksitewellness@tufts.edu](mailto:worksitewellness@tufts.edu) or mailed back to the HNRCA at 711 Washington St, Boston, MA 02111.
         4. Willingness to provide email ID and contact information to the commercial program facilitators to receive program materials and other feedback related to program progress.
         5. Willingness to complete outcome assessments and self-monitoring in order to track intervention effectiveness
         6. BMI ≥ 20 kg/m^2^ and < 50 kg/m^2^ at screening, if participant opts into the HPI arm
         7. BMI ≥ 25 kg/m^2^ and < 50 kg/m^2^ at screening, if participant opts into the weight loss arm
      2. Exclusion criteria:
         1. Employees who are temporary contract workers or employees who work remotely most of the time
         2. < 21 years of age
         3. Participation in a weight loss program or intensive wellbeing program at time of enrollment
         4. Pregnant or lactating (per self-reports, now or intended during study)
         5. Prior weight loss surgery or a medical complication that would prevent full participation
         6. Multiple severe dietary intolerances (e.g. gluten combined with dairy or meat) which would reduce intervention adherence
         7. Non-English speaking
         8. Individuals who have lost > 15 pounds in the past 6 months
         9. mobility limitations (inhibiting the ability to stand on one’s own or get on and off of the scale)
         10. Major diseases including active cancer or cardiovascular disease
         11. BMI < 25 kg/m^2^ or ≥ 50 kg/m^2^ at screening, for those that opt-in to the weight-loss group
         12. BMI <20 kg/m^2^ or ≥ 50 kg/m^2^ at screening, for those that opt-in to the HPI group

For those interested in the weight loss intervention, additional exclusion criteria are as follows:

1. Any condition that influences the ability to absorb food (e.g. inflammatory bowel disease or celiac disease)
2. Very active individuals (> 2 hours/day or >14 hours/week of vigorous activity).

We would like to note that there will be no exclusion of participants based on out of range blood values since the physician’s clearance form will serve as the reference document for inclusion in the study. Extreme values will be flagged and per the study physician’s recommendation participants will be sent a notification to see their primary care provider.

Non – English speaking participants will be excluded from participation as the proposed intervention programs and program related materials have yet to be translated into other languages and have yet to be designed or tested for non – English speaking populations. Furthermore we anticipate that since this is a worksite study most if not all employees will be English speaking

- - 1. Withdraw/Termination criteria: Participants will be withdrawn if they develop a condition that renders them ineligible after they sign consent including the period during which they are actively participating in the study. This includes women who self-report pregnancy, those whose health status changes affecting adherence to the diet, safety concerns for either the participant or the researchers, or outcome assessments (questionnaires) reveal that they have an exclusion criterion that they did not disclose at screening. Participants will be withdrawn from the study if they enroll in any other weight loss/intensive wellness program during the 18 month intervention. Furthermore the PIs with the consultation of the study physician may terminate or temporarily withdraw participants at their discretion if they feel that participant safety is of concern.

Participants may withdraw at any time for any reason. If participants decide that they wish to withdraw they will be provided a prorated stipend based on completion of study outcomes for the time point at which they withdraw and provided a stipend is identified at that time point.

If participants are deemed ineligible or withdraw at any time for any reason, any data collected up until this point in time will be retained throughout the duration of the study. We plan to discard this data 10 years after study completion. Retaining this data is necessary to report CONSORT chart details in any study publication(s).

- In addition as stated in the company agreement, “in the event of low employee participation (less than 50 individuals joining the interventions), Tufts may decide to exclude Company Name from the study” any eligible worksite that enrolls may be withdrawn by the Principal Investigator (s).

1. **Risk/benefit assessment:** This is a randomized controlled study that has minimal risk associated with the research activities for worksite participants.

The major potential risk associated with the study is considered to be breach of confidentiality. To address this, upon entry into the study, a coded study ID will be assigned to the study subject and biological materials and questionnaires to maintain confidentiality within the research setting. The signed consent forms data on paper forms will be kept in a locked file cabinet in the USDA Human Nutrition Research Center. Only the principal investigators and Study Coordinator will have access to the master code linking subject identifiers and their study ID. All investigators at Tufts undergo the required continuing recertification for ethical research by human subjects. Study investigators will see only de-identified data. The reviewing IRB is authorized to see fully identifiable data. Computer files and data collection forms will use the subject ID and will not be linked to protected information. The computers used to enter or store study data will be password protected. All data will be entered into StudyTRAX. Security features of StudyTRAX targets include password protection, restricted access to users based on role in the study, role-based access to features, access audit trails, and hosting services (e.g., firewall, secure sockets layer). Taken together, these features ensure access control, audit control, data integrity, user authentication, and transmission security. The research project will be set up in StudyTRAX using their standard procedure which ensures exported datasets are de-identified as defined in the HIPAA privacy regulation [45 C.F.R. §164.514 (b)(2)]. However, there is always the risk that confidentiality may be lost due to reasons beyond the control of study staff.

1. Physical risk: Minimal risk. Participants may experience some mild bruising or discomfort from the finger prick for the point of care assessments. The discomfort is normal and should dissipate. Excess weight loss may also be a potential risk if individuals continue to practice weight loss beyond the time period of the weight loss support group, but this is not recommended in the program. In addition, bloating, intestinal gas, or diarrhea has occasionally been reported with weight loss interventions as part of a change to a whole grain and higher-fiber diet. Participants in the HPI intervention may experience discomfort, during or following the exercise regimen that is provided as part of the training. Coaches will discontinue participants who report discomfort and it is anticipated that since participants have provided a physician’s clearance any discomfort following these sessions will be minimal and should be addressed with their primary care physician as needed. The Tanita Body Composition Analyzer is a scale with a bio-impedance analysis (BIA) function to measure body composition. To do this the device sends a very small charge through the body. The electric charge may interfere with electronic implanted devices. As a safety precaution, we will weigh participants with pacemakers or other implanted electronic devices using the weight-only feature of the scale. The BIA protocol and the data collection form include a question on use of pacemakers and other implanted electronic devices. Data collectors are instructed to elect the weight only feature to obtain weight for participants with these devices.
2. Psychological risk: The risk may be that no weight will be lost by some participants in the behavioral weight loss arms. Additionally, a potential risk is the discomfort associated with responding to questionnaires that may have sensitive information regarding weight and eating behavior.
3. Social risk: Knowledge of participation may not be completely confidential as there is a risk that coworkers are aware of employees who are enrollees in the wellness intervention programs. However, our experience is that the benefits (co-workers encouraging each other and other social support features) far out weight any potential risk and further we have not observed or have heard reports of negative effects.
4. Economic risk: It is possible that the participant may have to pay for a doctor’s visit if an in-person visit is required to obtain the physician clearance form. A small stipend is provided to cover such incidental expenses. No additional payment will be provided.

No compensation will be provided for missed work time for study activities however this will be planned with employee and management support to minimize/avoid missed work time and will be counted as credit rather than as time off.

For the diet arm of the study, class will only be for one hour during lunch or at another convenient time identified by the participants and facilitated by mutual agreement between the iDiet group leader and management. However, if the participants prefer a concurrent online session then intervention delivery will be provided using the online groups with flexibility for some in person groups. No compensation will be provided for missed work time for these classes. However, time required for study activities will be planned with employee and management support to minimize/avoid the impact of missed work time and will be counted as credit rather than as time off.

For the HPI program, a one-time initial workshop involving two and a half days will be required. No compensation will be provided, however time required for study activities will be planned with employee and management support to minimize/avoid the impact of missed work time and will be counted as credit rather than as time off. Please note that both weekend and weekday times will be offered for sign-up and if a participant absolutely cannot take a weekday off, they will have the option to choose weekend times, such as a Friday afternoon through the weekend.

1. Benefit of participation: The direct benefit is the opportunity of receiving a commercial diet or wellness program free of charge. This study has the potential for improved wellness for the participants in the areas of energy, health and weight related wellness. Furthermore, it is anticipated that findings from these studies will help to advance nutrition research.
2. **Specific methods and techniques used throughout the study**
3. **Laboratory tests:** We will measure fasted lipid panel including triglycerides, HDL, LDL and total cholesterol and glucose using the Alere Cholestech LDX® System which requires 40 microliters of blood. Hb A1c will also be measured using the Siemens DCA Vantage® which requires 1 microliter of blood. Research assistants will obtain capillary blood samples using a lancet. Measurements that are out of range (fasting blood glucose >126 mg/dl, TC >240 mg/dl, Hb A1c ≥6.5%) will be flagged in data given to participants, who will be recommended to follow up with their physician. (please note: as stated earlier physician clearance will be required prior to enrollment for all participants). Breakfast bars will be provided to all participants after overnight fasting blood work.

Laboratory tests will be completed at the worksite by study staff using paper data collection forms and will then be entered into the online, password protected database, using their numerical ID. This form has identifiers because the use of electronic de-identified data forms is not feasible due to the large number of participants moving through the measurement stations which could potentially cause errors in measurement recording if the study ID was the only identifier. These forms will be transported from the study site in a locked briefcase and will be kept in a locked file cabinet at the USDA Human Nutrition Research Center on Aging, as detailed in the confidentiality section below.

1. **Study Procedures**

Anthropometry: The research coordinators will take duplicate fasting measurements of weight in program participants to ±0.1 kg at baseline, 6, 12, and 18 months in all subjects using the Tanita TBF300A, self-calibrating scale with 200kg capacity. Participants will be asked to wear light clothing and no footwear. In addition, height will be measured in duplicate to ±0.1 cm at baseline using a portable stadiometer (Seca 214). Fasted waist and hip circumference will be measured in duplicate to ±0.3 cm at baseline, 6, 12, and 18 months in all subjects according to our standard procedures (Seca 201). Fasted blood pressure will be measured to the nearest 1 mmHg at baseline, 6, 12, and 18 months in all subjects using a validated automated monitor (3 measurements, 5 minutes apart after 5 minutes of quiet sitting, using the OMRON HEM-705CP Digital Blood Pressure Monitor). Measurements that are out of range (systolic BP >160 mmHg, diastolic BP >100 mmHg) will be flagged in data given to participants, who will be recommended to follow up with their physician (please note: as stated earlier physician clearance will be required prior to enrollment for all participants).

Anthropometric measurements will be completed at the worksite by study staff using paper data collection forms and will then be entered into the online, password protected database, using their numerical ID. This form has identifiers because the use of electronic de-identified data forms is not feasible due to the large number of participants moving through the measurement stations which could potentially cause errors in measurement recording if the study ID was the only identifier. These forms will be transported from the study site in a locked briefcase and will be kept in a locked file cabinet at the USDA Human Nutrition Research Center on Aging, as detailed in the confidentiality section below.

All study participants will be provided with pedometers (Yamax Digiwalker CW-701) to self-monitor their activity for one week at baseline, month 6, month 12 and month 18. Pedometers will be distributed and collected by study staff members at the beginning and end of each of these time points, and will be retained by the study staff at the end of the study. When pedometers are provided it will be emphasized that care and return are critical, however if they are lost there will be no penalty or replacement. Furthermore, they will be asked to self-report their activity for a week at baseline, month 6, month 12, and month 18 using the StudyTRAX database with electronic data capture that is built in to protect data confidentiality.

**Dietary Recalls:** Adherence to the energy intake and specific dietary recommendations of each intervention will be determined with 2 random 24 hour recalls by phone at three time points (baseline, during month 6 (active weight loss) and during month 18 (weight maintenance)), for a total of 6, 24 hour recalls throughout the entire study period. The recalls will be collected and analyzed by the Dietary Assessment Unit at Tufts, which provides a centralized and high standard dietary data service. The recalls will be analyzed for energy intake, macronutrient distribution, total fiber, energy density and meal patterns using Nutrition Data System for Research software version, 2012 developed by the Nutrition Coordinating Center (NCC), University of Minnesota, Minneapolis, MN. We have considerable experience with dietary records of different kinds and a history of lower underreporting than is typical (65). In addition, we will screen our dietary data prior to statistical analyses to eliminate implausible records (66, 67).

**Provided Foods:** Participants in the diet arm receiving the provided Yevo foods, will also be asked to check the log for meals consumed each day and record reasons for not consuming each meal such as satiety, monotony, palatability etc. Participants will be supplied with these foods monthly at their worksite for the first six months (equivalent to 2 meals/day during the first 12 weeks, and 1 meal/day for the following 12 weeks). No meals will be provided after this period, however weight maintenance in participants randomized to receive the meal will be examined for over the 18 month study duration, along with follow-up surveys to gauge participant enjoyment and opinion of provided Yevo foods.

**Grocery Store Receipts:** Participants in the diet arm will be asked to bring in all grocery store receipts for a 1-week period at baseline and 6 months to see what foods they are purchasing.

**Questionnaires:** Questionnaires will be completed to measure a variety of health-related variables as summarized in the Table below. A short battery of cognitive tests including for memory, attention and other related measures will be administered using standardized tests that are adapted for computer based testing by Dr. Miguel Alonso (Director of Nutritional Neuroscinece) at Beth Israel Deaconess Medical Center and Harvard Medical School, Boston, MA. A follow-up questionnaire will be distributed either in person, online or over the phone at months 12 and 18 to capture changes in medication, specifically for diabetes, blood pressure and cholesterol.

**Recruitment Day(s):** Recruitment Day will be used as an opportunity to encourage employee participation in the study among the twelve worksites that have been selected for the Weight Loss or Human Performance Institute interventions. We will work with HR to conduct Recruitment Days for each of the twelve worksites. We will hold additional Recruitment Day sessions on an as needed basis, or if the worksites feel that this would increase participation. This will be determined on an individual worksite basis. Brief informational sessions for all employees will also be held at the beginning of each Recruitment Day. Study staff will also be available, in-person, to answer specific questions regarding participation. In addition to using Recruitment Day as an opportunity to disseminate informational flyers and brochures explaining the mission of our intervention programs, both height and weight measurements will be obtained from those who sign up for the study. This form has identifiers because the use of electronic de-identified data forms is not feasible. As participants move through the various measurement stations before their eligibility is verified. Please see confidentiality section for how these forms will be stored and handled. For employees wishing to enroll in the wellness intervention programs, height and weight measurements obtained on Recruitment Day will be used to confirm eligibility based on the stated inclusion/exclusion criteria.

**Intervention goals and details for HPI and the iDiet**

| iDiet (iDiet) | |
| --- | --- |
| Primary goals | - *Goals*: Weight loss (1-2 lb/week), dietary energy reduction (**approximately** 500-1000 kcal/week) consumed in regular meals, encourage activity (150 minutes/week). Please note individualized calorie goals are used per program specifications, ranging from 1000 to 2500 calories depending on the weight of the participant. The minimum calorie target recommended for our smallest people will be 1000 calories/day, which may not be as great as a 500 calorie deficit, and participants are able to request a different menu level if they wish. The goal for physical activity is also a broad goal. Participants will be provided with pedometers and forms for tracking pedometer counts and other activities. If participants tracked activity levels do not meet program goals, counselors will suggest incremental strategies to increase activity weekly through brisk walking, lifestyle activities or other exercise of the participant’s choice. Physical activity will be recommended starting after Session 3 and will be emphasized as an important factor for preventing weight regain once subjects are in maintenance. Participants will not be excluded or withdrawn from the study if they do not meet this recommendation. - *Core strategies*: Group classes, behavioral skills training and materials (e.g. pedometer, family-friendly menu examples and a log for self-monitoring weight) to support study adherance. |
| Nutrition goals and strategies | - *Goals*: High fiber (>40 g/d), moderately high protein (26% energy), low glycemic load (48% energy with low GI carbs) - *Core strategies*: Use of provided calorie- and nutrient-controlled menus and recipes. General portion guidelines and self-monitoring for portion sizes |
| Behavioral goals and strategies | - *Goals*: Hunger supression and maintenance of satiety; changing food preferences - *Core strategies*: Adherence to specific, repetitive provided menus and cognitive restructuring exercises. Frequent meals, use of free foods, optional portion size monitoring; raising self-efficacy, exercise |

| Human Performance Institute (HPI) | |
| --- | --- |
| Primary goals | - *Goals*: The primary training goal is to improve individual performance and increase quality of life by aligning participants’ direction in life with how and where they spend their energy.  The integrated training model states that increased life engagement leads to enhanced performance through focusing personal resources on a defined purpose or mission. The intervention is based on two foundational models: 1) the Energy Management Model and 2) the Change Process Model. The Energy Management Model focuses on four domains of energy which includes the spiritual, mental, emotional and physical areas of energy. The Change Process Model focuses on the elements of purpose, truth, and action to help participants indentify their purpose in life. - *Core strategies*: Group classes, exercise sessions, behavioral skills training and materials (e.g. exercise sessions, 360˚ energy profile and a 90-day training mission) to support study adherance. |
| Nutrition goals and strategies | - *Goals*: Help individuals understand the role that nutrition plays in energy management, engagement and performance, with a particular focus on maintaining stable blood glucose levels. Training participants that listening to one’s own hunger and satiety signals can lead to ideal energy, mood, performance, and body composition. - *Core strategies*: Focus on the glycemic index of foods and snacks, becoming more aware of meal timing, and understanding “five-handfuls” per meal (two handfuls of fruit, two handfuls of grain, and one handful of protein). |
| Behavioral goals and strategies | - *Goals*: To have individuals find and define a purpose or direction in life, in order to identify motivating factors that can help participants make difficult changes. The rationale is to tap into what is called “intrinsic motivation”, which, research shows, will facilitate more sustainable behavior change (68). - *Core strategies*: Workshops to discuss one’s purpose, facing the truth, working through setbacks, using training logs and acountability plans to help support study adherance. |

1. **Usual standard therapy vs solely for research purposes:** N/A
2. **Confidentiality of body measurements:** Please note we have no tissue banking. All anthropometrics will be done in a private area using a screen and necessary privacy measures including for recording and storing the collected data.
3. **Subject Timeline**:

**Table 1: Overview of Assessments and Assessment Schedule (in weeks):**

|  | **Intervention Worksites – HPI and iDiet Groups*** | **Control Worksites – Future HPI, and iDiet Groups*** |
| --- | --- | --- |
| **Measurements** |  |  |
| **Weight at site** | **0, 3, 6, 9, 12, 15, 18** | **0, 3, 6** |
| **Blood Pressure, Fasting lipid panel and Glucose** | **0, 6, 12, 18** | **0, 6** |
| **Waist and Hip Circumference** | **0, 6, 12, 18** | **0, 6** |
| **% Body fat** | **0, 3, 6, 12, 18** | **0, 3, 6** |

**Table 2: Overview of Questionnaires at Each Time point (in weeks):**

| **Questionnaires** | **Intervention Worksites – iDiet, and HPI Groups*** | **Control Worksites – Future iDiet, and HPI Groups*** |
| --- | --- | --- |
| **Demographics & Brief Medical History** | **0** | **0** |
| **Rand Quality of Life (SF-36)** | **0, 3, 6, 12, 18** | **0, 3, 6** |
| **IPAQ Physical Activity Questionnaire – Short form** | **0, 6, 12, 18** | **0, 6** |
| **Cravings Questionnaire** | **0, 6, 12, 18** | **0, 6** |
| **Three Factor Eating Questionnaire – R51** | **0, 6, 12, 18** | **0, 6** |
| **Food Preferences Questionnaire** | **0, 6, 12, 18** | **0, 6** |
| **MOS Sleep Questionnaire** | **0, 6, 12, 18** | **0, 6** |
| **Work Productivity and Impairment Scale (WPAI)** | **0, 6, 12, 18** | **0, 6** |
| **Ryff Purpose in Life Scale** | **0, 3, 6, 12, 18** | **0, 3, 6** |
| **Morningness Eveningness Questionnaire** | **0** | **0** |
| **Center for Epidemiologic Studies Depression Scale**  **(CES-D)** | **0, 6, 12, 18** | **0, 6** |
| **Profile of Mood States (POMs)** | **0, 6, 12, 18** | **0, 6** |
| **Cognitive Tests (computer based)** | **0, 6, 12, 18** | **0, 6, 12, 18** |

1. **Assessment of Participant Safety and Development of a Data and Safety Monitoring Plan (DSMP)**

A Data and Safety Monitoring Plan will be implemented to ensure the safety of the participants, as well as the validity and the integrity of the data. Dr. Das with the help of the study physician and research coordinators will monitor all aspects of safety. A safety monitoring log will be developed and participants will be asked to record signs, symptoms and both serious and non-serious adverse events. Research assistants will be instructed to report any adverse events that occur during research activities to the PI as soon as possible. Both serious and non-serious events will be documented. An event will be considered serious if it results in death, is life-threatening, requires inpatient hospitalization, or results in a persistent or significant disability/incapacity, or might require medical or surgical intervention to prevent one of these outcomes. Non-serious events are defined as conditions that may be unpleasant to the participant, such as sore muscles, but do not require termination of participation. Any participant who experiences an adverse event has the option to continue participation in the study, if she so chooses and if it is safe and appropriate.

We do not consider excessive weight loss to be a risk because we do not anticipate this and will monitor for this as we have in our previous trials (34, 35). Further, the study physician will review the weight loss of individual participants on a quarterly basis. In the event that participants report symptoms or concerns about excess weight loss, or lightheadedness, intensive exercising, or if development of disordered eating is suspected, the study physician will review reports; he will then assess the participant by phone or in person to determine if appropriate referral to personal health care providers and/or exclusion from the trial is indicated. In the unlikely event that there are individuals whose BMI falls below 21.0 kg/m^2^ and still want to lose more weight, study physician will communicate by phone or in person to recommended no further weight loss. Individuals who do not comply with this recommendation will be excluded from further trial participation.

**Reporting timeframe for SAEs and AEs:**

Investigators will promptly report unexpected adverse events, serious adverse events, unanticipated problems involving risk to subjects or others, and deviations to the IRB. Serious adverse events will be reported to the Tufts IRB within five business days. Non-serious adverse events will be reported to the Tufts IRB in a summary report, specifying the nature and frequency of the adverse events, at the time of continuing review. Any non-serious adverse events that are deemed an unanticipated problem will be reported to the IRB as for a serious adverse event. Unanticipated problems are defined as: 1) The nature, severity, or frequency is unexpected for the participant population or research activities; 2) related or possibly related to participation in the research; and 3) an event indicating that the research may place the participant or others at a greater risk of harm then was previously recognized.

The study physician will review all reports of AEs at least monthly. The study physician is the Medical Director of the inpatient Metabolic Research Unit at Tufts and has extensive experience in the safe conduct of human research procedures to protect human subjects. If adverse event monitoring suggests that events are occurring with greater frequency or severity than initially expected, the PI will submit a report of an Unanticipated Problem to the IRB, with an amendment if a modification of the study design is indicated.

In addition, all unanticipated problems involving risk to subjects or others, serious adverse events related to participation in the study and subject deaths related to participation in the study will be promptly reported by phone to the IRB.

**Accountability procedures as they relate to drugs, devices, and data:**

There are no drugs or devices in this study. Concerning computer files and data collection forms, these will use the subject ID and will not be linked to protected information. The computers used to enter or store study data will be password protected. All data will be entered into StudyTRAX. Security features of StudyTRAX targets include password protection, restricted access to users based on role in the study, role-based access to features, access audit trails, and hosting services (e.g., firewall, secure sockets layer). Taken together, these features ensure access control, audit control, data integrity, user authentication, and transmission security. The research project will be set up in StudyTRAX using their standard procedure which ensures exported datasets are de-identified as defined in the HIPAA privacy regulation [45 C.F.R. §164.514 (b)(2)].

1. **Subject Participation**
2. **Recruitment** A multi-staged screening process will be used to identify 12 suitable worksites for the study. We will contact worksites withign the greater Boston area including those which have previously expressed interest or with whom Tufts has corporate affiliations with to find 12 worksites who are eligible and interested in doing the study. The 12 shortlisted and eligible worksites, will be invited to enroll. Companies and government institutions in the professional sector, and located in the Greater Boston area who report 300 or more employees will be contacted by land mail and/or email with an initial information letter describing the study and highlighting the benefits of participation. Companies with greater than 900 employees will potentially be divided into 2 or more subunits to make up the 12 worksites. The initial information letter will invite interested worksites in applying to the intervention program. We will send follow-up information with further details of study participation to the companies who respond to the initial mailing. Study staff will meet in person or talk with HR personnel/company representative about the process of being considered for participation. HR personnel/company representative will then mail and/or email employees with the informational email and details about recruitment & screening day.
3. **Registration:** This study will be enrolled as a clinical trial on clintrials.gov.
4. **Screening Interview/Questionnaire:**

Those interested in participating will be screened on **Recruitment** Day. They will first be administered the screening ICF, and the screening will involve a questionnaire on exclusions and measurements of weight and height for calculation of BMI and the option to choose the diet or the HPI arm of the intervention. If the individual is eligible they will then be administered with the informed consent form (ICF) for baseline assessments. The **Recruitment** Days, screening and baseline measurements will be conducted by members of the Tufts team (study coordinators, research staff and investigators) depending on availability.

Randomization of worksites to the intervention or control condition will be done following the completion of baseline assessments. Once worksites are randomized to receive the intervention or weight-listed control condition, participants within the intervention worksite will be assigned to the intervention arm that they opted into at screening i.e. HPI or diet arm. However, those who had expressed an interest in the

iDiet will be further randomized to receive Yevo foods or a gift card. Once randomization has occurred, participants will be given the consent form to read and sign prior to enrollment in the study.

Any questions or concerns will be addressed by a study team member prior to signing of the ICF. Copies of the signed ICF will be given to the study participants. Enrollment of all participating sites and employees will occur over a 6 month period during the first year.

1. **Transportation:** No transportation will be provided for this study since all support and assessments are provided and conducted in the workplace.
2. **Informed consent process:** Eligibility for inclusion of a worksite is multistep and is summarized in the protocol (Section III, parts C and D).
   - 1. Informed Consent for Initial Web Survey For All Employees at Worksite
     2. HR Company Agreement Form
     3. Informed Consent for Worksite **Recruitment** Day and Screening
     4. Informed Consent for Baseline Assessments For All Employees who have expressed an interest to join an intervention group at Worksite
     5. Informed Consent for:
3. iDiet Intervention
4. Human Performance Institute Intervention
5. Control group
6. **Location:** Most of the study will be done at the worksite or online based on the intervention arm. All of the physical measurements will be done at the worksite of the participant. For those involved in the HPI intervention, there will be sessions at an alternative commutable location that is off site, at their worksite’s gym or at a nearby gym affiliated with the given worksite.
7. **Personnel**

Dr. Sai Krupa Das (Principal Investigator (IRB)) will be responsible for overall management of the study and oversight, IRB reporting and management of the study outcomes. She will participate in study- wide investigator and sub-committee meetings and corporate interactions.

Dr. Susan Roberts (Co-PI) will be responsible for oversight of the weight loss intervention arms and participate in study- wide investigator and sub-committee meetings and corporate meetings and will provide strategies essential to the successful of diet interventions.

Dr. Cheryl Gilhooly (Co-Investigator) will be responsible for monitoring dietary recalls as well as participating in the study-wide investigator meetings providing advice and strategies necessary for a successful diet data collection.

Dr. Edward Saltzman (Study Physician) will be responsible for safety oversight of the study including oversight of adverse events and monitoring and advise in preparing reports on serious adverse events for the Tufts IRB. He will also discuss any necessary safety issues with study participants. He is the Medical Director of human studies at the Jean Mayer USDA Human Nutrition Research Center on Aging (HNRCA) at Tufts University and has substantial experience in the supervision of medical safety. As the study physician, he will also participate in the study-wide investigator and sub-committee meetings.

Gail Rogers (Senior Statistician) will provide oversight and management of all data analysis, working closely with the senior data manager. She will also participate in all study-wide investigator and sub-committee meetings.

Kara Livingston (Senior Data Manager) will provide oversight and management of StudyTrax, data cleaning and readying files for analysis and work closely with the statistician. She will also participate in all study-wide investigator and sub-committee meetings.

Taylor Vail (Senior Research Coordinator) will be serving as the research coordinator, coordinating study measurements and managing study flow. She will also participate in all study-wide investigator and sub-committee meetings.

Meghan Chin (Research Assistant) will be responsible for the diet arm, collecting outcomes for all participants and assisting with study flow and data management when needed.

Please note that the worksite wellness intervention programs, iDiet, and HPI are commercial intervention programs with professionally trained wellness coaches that will deliver the respective interventions.

**Consultants:** Dr. Putzke and his staff at ScienceTRAX will work closely with Dr. Das, Ms. Vail and the data manager in the development and deployment of the StudyTRAX system in accordance with study requirements, ensuring all data sets are de-identified as defined in the HIPAA privacy regulation, and will facilitate data hosting and providing files ready for analysis.

**Confidentiality:** Upon entry into the study, a coded study ID will be assigned to the study participants and also for questionnaires to maintain confidentiality within the research setting. The signed consent forms and any paper data collection forms will be transported from the study site in a locked briefcase and will be kept in a locked file cabinet at the USDA Human Nutrition Research Center. Only the principal investigators and Tufts Study Coordinator will have access to the master code linking subject identifiers and their study ID. The other study team members will have restricted or limited access to components of the data that are essential for intervention delivery or will only have access to de-identified data. Participants will also have access to their own weight data (iDiet) and all biometric data (HPI) and are encouraged to share it with their wellness coaches as they see fit. The master file with study ID code will be destroyed 10 years after the study is complete. Signed consent forms and screening questionnaires that are collected at study sites will be kept in a number lock briefcase at collection sites and during transport back to Tufts University. Only the principal investigators and Tufts study coordinator will have access to unlock the file cabinet. The Tufts Study Coordinator will keep the key for the file cabinet. After 10 years all hard copy data will be shredded. Additionally, data will be kept in a secured protected drive at Tufts for 10 years after closure of the study unless the IRB grants longer. All investigators at Tufts undergo the required continuing recertification for ethical research by human subjects. Computer files and data collection forms will use the subject ID and will not be linked to protected information. The computers used to enter or store study data will be password protected. All data will be entered into StudyTRAX. Security features of StudyTRAX targets include password protection, restricted access to users based on role in the study, role-based access to features, access audit trails, and hosting services (e.g., firewall, secure sockets layer). StudyTrax uses the data center services at Rackspace, a premier hosting company. Characteristics of the Rackspace data center include a secure biometric access that is monitored 24/7 by closed circuit cameras; no public access; large-scale HVAC systems; continuous uninterrupted power supply systems; diesel engine backup on site and enterprise class routing equipment. In addition, there is a dedicated managed Cisco router firewall; all server requests are transmitted over SSL using 256-bit encryption; redundant array of independent disks (RAID) level 5, nightly system; database backups and nightly database integrity checks. StudyTrax database will not include computer IP addresses. StudyTrax will be notified that investigators are done with the data and have finished the study. At that time, StudyTrax will delete all participant data and audit logs. Taken together, these features ensure access control, audit control, data integrity, user authentication, and transmission security. The research project will be set up in StudyTRAX using their standard procedure which ensures exported datasets are de-identified as defined in the HIPAA privacy regulation [45 C.F.R. §164.514 (b)(2)].

1. **Collaboration:** This project is collaboration between researchers at the JM USDA HNRCA at Tufts University, Johnson and Johnson’s Wellness and Prevention Division (J&J WPN), and Yevo International, LLC (Yevo Intl).

For those employees participating in the HPI program, their first and last name as well as their work email address will be provided to J&J-WPN identified, Performance Programs Inc. (a third party provider) that will disseminate a package of information, and a small battery of questionnaires directly to the participant. This data will be returned to J&J’s WPN coaches and will be used during the 2.5 day workshop. Similarly, participants’ information such as first and last names and email address will have to be shared with iDiet so these commercial programs can be effectively communicated and disseminated to the participants.

Most study data is being entered in a secure website which will store the data under encrypted numbers. Only summary report data will be shared with our collaborating investigators at J&J WPN and Yevo Intl. However, no data files or datasheets with identifiers will be transacted or shared between Tufts University and J&J WPN (except participants may share their own data with the professional coaches for feedback) or Yevo Intl. Companies will not be providing information for this study. All outcome measures required for the study will be obtained by Tufts. Should we identify any information that would be useful and can be provided by the companies we will request and obtain IRB permission prior to transfer and use of such information.

For reports, presentations and such activities aggregate data will be provided or shared with no reference to any identifiers.

1. **Alternatives:** The alternative to participating in this study is not to participate. Additionally, since these are commercial programs, another alternative is to participate in these programs on their own time.
2. **How new information will be conveyed to the study participant and how it will be documented:** Participants will be informed in writing of any significant new findings developed during the course of participation in this research that may have a bearing on their willingness to continue in this study. We will provide each participant with a summary of trial results after the primary paper for the study is published.
3. **Payment, including a prorated plan for payment**

Participants will receive up to $75 over the course of the study, for completing the assessments outlined at each time point (Table 1). Participants will receive this amount as $25 for doing all 6 month testing, $25 for doing all of month 12 testing, and $25 for doing all of month 18 testing. These amounts will be prorated and given to participants based on completion of testing at each time point and if participants withdraw, or are withdrawn from the study for any reason. If institutional policy dictates that employees are not allowed to receive such stipends then such policies will override the provision of stipends as stipulated for each time point.

For diet intervention participants randomized to the voucher group, vouchers of $63 will be given at baseline and weeks 4, 8, 12, and 16, 20 the time of food distribution, for a total of $378 in gift card vouchers.

1. **Payment for research-related injury:** Subjects will be encouraged to see their primary care physician, however no monetary compensation for injury will be offered.
2. **Outcomes**

The primary outcomes are a change in measured weight from baseline to 18 months in iDiet participants, and a statistically significant improvement in energy status for HPI participants and baseline to 6 months in comparisons with the controls. Additional outcomes include: cardiometabolic risk factors, quality of life, employee productivity, purpose in life, measured dietary intake and cravings, satiation and satisfaction of food provisions, as well as changes in physical activity. Further outcomes include changes in weight, blood pressure employee energy and productivity in non-participating employees at the intervention worksites. Differences in overall mean values between the intervention participants and controls, as well as overall mean values between interventions will be compared using *t*-tests and proportions will be compared using *χ*2 analyses.

1. **Tissue banking considerations:** There will be no tissue samples taken as part of this study and no tissue banking is planned or anticipated.

**Appendix I: Vulnerable Populations**

- No vulnerable populations will be studied.

**Literature Review**

1. Hertz RP, Unger AN, McDonald M, Lustik MB, & Biddulph-Krentar J. The impact of obesity on work limitations and cardiovascular risk factors in the U.S. workforce. Journal of Occupational Environmental Medicine. 2004; 46(12):1196-1203.
2. Boles M, Pelletier B, & Lynch W. The relationship between health risks and work productivity. Journal of Occupational Environmental Medicine. 2004;46(7):737-745.
3. Rodbard HW, Fox KM, & Grandy S. Impact of obesity on work productivity and role disability in individuals with and at risk for diabetes mellitus. American Journal of Health Promotion. 2009;23(5):353-360.
4. Ostbye T, Dement JM, & Krause KM. Obesity and workers’ compensation. JAMA Internal Medicine. 2007;167:766-773.
5. Pollack KM, Sorock GS, Slade MD, Cantley L, Sircar K, Taiwo O, & Cullen MR. Association between body mass index and acute traumatic workplace injury in hourly manufacturing employees. American Journal of Epidemiology. 2007;166(2):204-211.
6. Mokdad, AH, Bowman BA, Ford ES, Vinicor F, Marks JS, & Koplan JP. The continuing epidemics of obesity and diabetes in the United States. Jama. 2001; 286(10): p. 1195-200.
7. Ogden CL, Carroll MD, Brian KK, Flegal KM. Prevalence of childhood and adult obesity in the United States, 2011-2012. JAMA. 2014; 311(8):806-14.
8. Levi J, Segal LM, Laurent RS, Rayburn J. F as in fat: How obesity threatens america’s future. Robert Wood Johnson Foundation. 2012; 1-123.
9. Sabinsky, MS, Toft U, Raben A, &Holm L. Overweight men's motivations and perceived barriers towards weight loss. Eur J Clin Nutr. 2007; 61(4):526-31.
10. Zunker C, Cox TL, Wingo BC, Knight BN, Jefferson WK, & Ard JM. Using formative research to develop a worksite health promotion program for African American women. Women Health., 2008; 48(2):189-207.
11. Must A, Spadano J, Coakley EH, Field AE, Colditz G, & Dietz WH. The disease burden associated with overweight and obesity*.* JAMA. 1999; 282:1523-29.
12. Larsson, B., P. Bjorntorp, and G. Tibblin. The health consequences of moderate obesity. Int J Obes. 1981; 5(2):97-116.
13. Burton WN, Chen CY, Schultz AB, & Edington DW. The economic costs associated with body mass index in a workplace. J Occup Environ Med. 1998; 40(9): 786-792.
14. Thompson, Estimated economic costs of obesity to U.S. business. Am J Health Promot. 1998; 13(2):120-27.
15. Tucker, L.A. and A.G. Clegg, Differences in health care costs and utilization among adults with selected lifestyle-related risk factors. Am J Health Promotion. 2002; 16(4):225-33.
16. Engbers L, van Poppel M, Chin A, Paw M, & van Mechelen W. The effects of a controlled worksite environmental intervention on determinants of dietary behavior and self- reported fruit, vegetable and fat intake. BMC Public Health. 2006; 6(1):253.
17. Hennrikus, D.J. and R.W. Jeffery, Worksite intervention for weight control: a review of the literature. Am J Health Promot, 1996. 10(6): 471-498.
18. Hoke CN, Franks S. Work site, physician’s office, or medical university clinic: The effect of setting on success in a multidisciplinary weight-loss program. Eating Behaviors. 2002;3(1):93-100.
19. Katz DL, O'Connell M, Yeh MC, Nawaz H, Njike V, Anderson LM, et al. Public health strategies for preventing and controlling overweight and obesity in school and worksite settings: a report on recommendations of the Task Force on Community Preventive Services. MMWR Recomm Rep. 2005;7;54(54):1-12.
20. Kumanyika, S., et al., Obesity prevention: the case for action. Int J Obes Relat Metab Disord., 2002. 26: 425-436.
21. Kumanyika S, Brownson RC, Yancey A, Pronk N, Cole B. Workplace Approaches to Obesity Prevention. Handbook of Obesity Prevention: Springer US; 2007. p. 317-47.
22. Kwak L, Kremers SPJ, Visscher TLS, van Baak MA, Brug J. Behavioral and cognitive effects of a worksite-based weight gain prevention program: The NHF-NRG in balance- project. J Occup Environ Med. 2009; 51(12):1437-46.
23. Mills PR, Kessler RC, Cooper J, & Sullivan S. Impact of a health promotion program on employee health risks and work productivity. American Journal of Health Promotion. 2007;22(1):45-53.
24. Morgan PJ, Collins CE, Plotnikoff RC, Cook AT, Berthon B, Mitchell S, et al. Efficacy of a workplace-based weight loss program for overweight male shift workers: The Workplace POWER (Preventing Obesity Without Eating like a Rabbit) randomized controlled trial. Preventive Medicine. 2011; 52:317-325.
25. Rigsby A, Gropper DM, Gropper SS. Success of women in a worksite weight loss program: Does being part of a group help? Eating Behaviors. 2009;10(2):128-30.
26. Anderson LM, Quinn TA, Glanz K, Ramirez G, Kahwati LC, Johnson DB, et al. The effectiveness of worksite nutrition and physical activity interventions for controlling employee overweight and obesity: A systematic review. Am J Prev Med. 2009;37(4):340-57.
27. Ni Mhurchu C, Aston L, Jebb S. Effects of worksite health promotion interventions on employee diets: a systematic review. BMC Public Health. 2010;10(1):1-7.
28. Beresford SAA, Thompson B, Feng Z, Christianson A, McLerran D, Patrick DL. Seattle 5 a day worksite program to increase fruit and vegetable consumption.Preventive Medicine. 2001;32(3):230-8.
29. Glasgow RE, Terborg JR, Hollis JF, Severson HH, Boles SM. Take heart: results from the initial phase of a work-site wellness program. Am J Publ Health. 1995;85(2):209-16.
30. Glasgow R, Terborg J, Stryker L, Boles S, Hollis J. Take Heart II, Replication of

a Worksite Health Promotion Trial. J Behav Med. 1997;20(2):143 - 59.

1. Tilley B, Glanz K, Kristal A. Nutrition intervention for high-risk auto workers:

results of the Next Step Trial. Prev Med. 1999; 28:284-92.

1. Biener L, Glanz K, McLerran D. Impact of the Working Well Trial on the worksite smoking and nutrition environment. Health Educ Behav. 1999; 26:478-94.
2. Emmons K, Linnan J, Shadel W, Marcus B, Abrams D. The working healthy project: A worksite health-promotion trial targeting physical activity, diet and smoking. J Occup Med Environ Health. 1999;41(7):545 - 55.
3. Jeffery RW, Wing RR, Thorson C, Burton LR, Raether C, Harvey J, Mullen M. Strengthening behavioral interventions for weight loss: A randomized trial offood provision and monetary incentives. 1993; 6:1038-1045.
4. Wing RR & Jeffery RW. Food provision as a strategy to promote weight loss. 2001; 9(4) 271S-275S.
5. Salinardi TC, Batra P, Roberts SB, Urban LE, Robinson LM, Pittas AG, et al. Lifestyle intervention reduces body weight and improves cardiometabolic risk factors in worksites. Am J Clin Nutr. 2013; 97(4):667-76.
6. Batra P, Das S, TC S, Robinson L, Saltzman E, Scott T, et al. Relationship of cravings with weight loss and hunger: Results from a 6 month worksite weight loss intervention. Appetite. 2013; In Press.
7. Gorin AA, Wing RR, Fava JL, Jakicic JM, Jeffery R, West DS, et al. Weight loss treatment influences untreated spouses and the home environment: evidence of a ripple effect. Int J Obes. 2008; 32(11):1678-84.
8. Ahern AL, Olson AD, Aston LM, & Jebb SA. Weight Watchers on prescription: An observational study of weight change among adults referred to Weight Watchers by the NHS. BMC Public Health. 2011; 11(434).
9. Dansinger ML, Gleason JA, Griffith JL, Selker HP, & Schaefer EJ. Comparison of the Atkins, Ornish, Weight Watchers, and Zone diets for weight loss and heart disease risk reduction. JAMA. 2005; 293:43-53.
10. Wadden TA, Neiberg RH, Wing RR, Clark JM, Delahanty LM, Hill JO, et al. Four-year weight losses in the Look AHEAD study: factors associated with long-term success. Obesity. 2011; 19(10):1987-98.
11. Funk KL, Stevens VJ, Appel LJ, Bauck A, Brantley PJ, Champagne CM, et al. Associations of internet website use with weight change in a long-term weightloss maintenance program. J Med Internet Res. 2010; 12(3).
12. Kruger J, Blanck HM, Gillespie C. Dietary and physical activity behaviors among adults successful at weight loss maintenance. Int J Behav Nutr Phys Act. 2006;3:17.
13. Hindle L, Carpenter C. An exploration of the experiences and perceptions of people who have maintained weight loss. J Hum Nutr Diet. 2011; 24(4):342-50.
14. Linde JA, Jeffery RW, French SA, Pronk NP, Boyle RG. Self-weighing in weight gain prevention and weight loss trials. Ann Behav Med. 2005;30(3):210-6.
15. Pasman WJ, Saris WH, Westerterp-Plantenga MS. Predictors of weight maintenance. Obes Res. 1999; 7(1):43-50.
16. Wing RR, Papandonatos G, Fava JL, Gorin AA, Phelan S, McCaffery J, et al. Maintaining large weight losses: the role of behavioral and psychological factors. J Consult Clin Psychol. 2008; 76(6):1015-21.
17. Meffert C, Gerdes N. Program adherence and effectiveness of a commercial nutrition program: the metabolic balance study. J Nutr Metab. 2010;197656:21.
18. McGuire MT, Jeffery RW, French SA, Hannan PJ. The relationship between restraint and weight and weight-related behaviors among individuals in a community weight gain prevention trial. Int J Obes Relat Metab Disord. 2001; 25(4):574-80.
19. Larsen TM, Dalskov SM, van Baak M, Jebb SA, Papadaki A, Pfeiffer AF, et al. Diets with high or low protein content and glycemic index for weight-loss maintenance. N Engl J Med. 2010; 363(22):2102-13.
20. Gilhooly CH, Das SK, Golden JK, McCrory MA, Rochon J, DeLany JP, et al. Use of cereal fiber to facilitate adherence to a human caloric restriction program. Aging Clin Exp Res. 2008;20(6):513-20.
21. Phelan S, Liu T, Gorin A, Lowe M, Hogan J, Fava J, et al. What distinguishes weight- loss maintainers from the treatment-seeking obese? Analysis of environmental, behavioral, and psychosocial variables in diverse populations. Ann Behav Med. 2009; 38(2):94-104.
22. Klem ML, Wing RR, McGuire MT, Seagle HM, Hill JO. A descriptive study of individuals successful at long-term maintenance of substantial weight loss. Am J Clin Nutr. 1997;66(2):239-46.
23. McGuire MT, Wing RR, Klem ML, Hill JO. Behavioral strategies of individuals who have maintained long-term weight losses. Obes Res. 1999; 7(4):334-41.
24. Raynor HA, Van Walleghen EL, Bachman JL, Looney SM, Phelan S, Wing RR. Dietary energy density and successful weight loss maintenance. Eat Behav. 2011; 12(2):119-25.
25. Westerterp-Plantenga MS, Lejeune MP, Nijs I, van Ooijen M, Kovacs EM. High protein intake sustains weight maintenance after body weight loss in humans. Int J Obes Relat Metab Disord. 2004; 28(1):57-64.
26. Lejeune MP, Kovacs EM, Westerterp-Plantenga MS. Additional protein intake limits weight regain after weight loss in humans. Br J Nutr. 2005;93(2):281-9.
27. Nackers LM, Ross KM, Perri MG. The association between rate of initial weight loss and long-term success in obesity treatment: does slow and steady win the race? Int J Behav Med. 2010; 17(3):161-7.
28. Fabricatore AN, Wadden TA, Moore RH, Butryn ML, Heymsfield SB, Nguyen AM. Predictors of attrition and weight loss success: Results from a randomized controlled trial. Behav Res Ther. 2009; 47(8):685-91.
29. McGuire MT, Wing RR, Klem ML, Seagle HM, Hill JO. Long-term maintenance of weight loss: do people who lose weight through various weight loss methods use different behaviors to maintain their weight? Int J Obes Relat Metab Disord. 1998; 22(6):572-7.
30. Wadden TA, Butryn ML, Byrne KJ. Efficacy of lifestyle modification for long-term weight control. Obes Res. 2004;12:151S-62S.
31. Elfhag K, and S. Rossner. Who succeeds in maintaining weight loss? A conceptual review of factors associated with weight loss maintenance and weight regain. Obes Rev. 2005;6(1):67-85.
32. Weiss EC, Galuska DA, Kettel Khan L, Gillespie C, & Serdula MK. Weight regain in U.S. adults who experienced substantial weight loss, 1999-2002. Am J Prev Med. 2007; 33(1):34-40.
33. Buysse DJ, Reynolds,C.F., Monk,TH, Berman,SR, & Kupfer, DJ. The Pittsburgh Sleep Quality Index (PSQI): A new instrument for psychiatric research and practice. Psychiatry Research. 1989; 28(2):193-213.
34. Trabnulsi, J, and DA Schoeller. Evaluation of dietary assessment instruments against doubly labeled water, a biomarker of habitual energy intake. AJP-Endocrinology Metabolism. 2001; 281:E891-99.
35. Black, AE. Critical evaluation of energy intake using the Goldberg cut-off for energy intake:basal metabolic rate. A practical guide to its calculation, use and limitations. International Journal of Obesity. 2000; 24:1119-30.
36. McCrary, MA, Hajduk, CL, & Roberts SB. Procedures for screening out inaccurate reports of dietary energy intake. Public Health Nutrition. 2002; 5(6A):873-882.
37. Ryan RM, and EL Desi. Self-determination theory and the facilitation of intrinsic motivation, social development, and well-being. American Psychologist. 2000; 55(1):68-78.
