# Supplementary material for: Effectiveness of an Energy Management Training Course on Employee Well-Being: A Randomized Controlled Trial
Source: Am J Health Promot. 2018 May 28;33(1):118–30. doi: 10.1177/0890117118776875 (PMC7323760; doi:10.1177/0890117118776875)
Supplement: Supplemental Material, Permission_Request-Corporate_Athlete_Outline1_(1) - Effectiveness of an Energy Management Training Course on Employee Well-Being: A Randomized Controlled Trial [file Permission_Request-Corporate_Athlete_Outline1_(1).pdf]

Johnson & Johnson Health and Wellness Solutions, Inc. ("JJHWS")

LIMITED USE AGREEMENT - PRINTED MATERIAL

DATE: 4/5/2018

TO:

Requester: Dr. Sai Krupa Das

Title: Scientist I

Institution: Jean Mayer USDA Human Nutrition Research Center on Aging at Tufts Univeristy

Address: 711 Washington Street
Boston, MA 02111

Phone Number: (617) 556-3133

Email: Sai.das@tufts.edu

For the purpose of inclusion in the following article (the "Article") and publication in the following journal (the "Journal", the Article and the Journal are collectively referred to as the "Work"):

TITLE OF THE ARTICLE*: Effectiveness of an Energy Management Training Course on Employee Well-being: A Randomized Controlled Clinical Trial*

TITLE OF THE JOURNAL: *The American Journal of Health Promotion, published by SAGE Publications, Inc.*

we are providing written permission to reprint the below material ("Material"), a copy of which is attached to this agreement:

[INSERT TITLE OR DESCRIPTION OF PIECE TO BE INCLUDED AND ATTACH COPY]
J&J HPI 2.5 Day Course outline

The following is agreed upon by you as the requester:

- Permission, via a limited license, is granted to Requester to include the Material in the Work only to the extent that JJHWS has the right to grant such permission. No guarantee is given or made that JJHWS has sufficient rights for the Requester to use the Material as desired by the Requester.
- Permission is for use only in the Work listed above. This permission includes the non-exclusive right throughout the world, in all formats and media now known or later developed, for the Journal owner to: reproduce, reprint, distribute, transmit and display the Material solely in connection with and exactly as it appears within the Article.
- Material will not be modified or redrawn in any way.
- The Work shall not portray the Material in a disparaging, negative or derogatory manner.
- JJHWS reserves the right to revoke permission at any time upon written notice.
- Requested changes (if any) to the text accompanying the Material will be made. Otherwise, Material may not be used.
- The permission granted herein may not be assigned or licensed to any other party without the prior written consent of JJHWS.
- All rights not expressly granted herein are reserved by JJHWS.

Please sign below and return this form via email to _smason5@its.jnj.com. Once signed by a JJHWS representative, this form will be returned to you. Permission will take effect once both signatures are in order.

Sai Krupa Das

Requester Signature

4/5/2018

Date

Johnson & Johnson Health and Wellness Solutions, Inc.
Representative

Name: ______________________________

JO ANN SWASEY

Date

Digitally signed by JO ANN SWASEY
DN: c=US, o=JNJ, ou=Subscribers, 0.9.2342.19200300.100.1.1=32281, cn=JO ANN SWASEY
Reason: I am approving this document.
Date: 2018.04.09 13:18:12 -04'00'
Adobe Reader version: 11.0.10
